# *“I fought my entire way”*: Experiences of declining maternity care services in British Columbia

**DOI:** 10.1371/journal.pone.0252645

**Published:** 2021-06-04

**Authors:** P. Mimi Niles, Kathrin Stoll, Jessie J. Wang, Stéphanie Black, Saraswathi Vedam

**Affiliations:** 1 Meyers College of Nursing, New York University, New York, NY, United States of America; 2 Birth Place Lab, Department of Family Practice, University of British Columbia, Vancouver, British Columbia, Canada; 3 MD Undergraduate Program, Faculty of Medicine, University of British Columbia, Vancouver, British Columbia, Canada; Liverpool School of Tropical Medicine, UNITED KINGDOM

## Abstract

**Background:**

The 2016 WHO Standards for improving quality of maternal and newborn care in health facilities established patient experience of care as a core indicator of quality. Global health experts have described loss of autonomy and disrespect as mistreatment. Risk of disrespect and abuse is higher when patient and care provider opinions differ, but little is known about service users experiences when declining aspects of their maternity care.

**Methods:**

To address this gap, we present a qualitative content analysis of 1540 written accounts from 892 service users declining or refusing care options throughout childbearing with a large, geographically representative sample (2900) of childbearing women in British Columbia who participated in an online survey with open-ended questions eliciting care experiences.

**Findings:**

Four themes are presented: 1) Contentious interactions: *“I fought my entire way”*, describing interactions as fraught with tension and recounting stories of “fighting” for the right to refuse a procedure/intervention; 2) Knowledge as control or as power: *“like I was a dim girl”*, both for providers as keepers of medical knowledge and for clients when they felt knowledgeable about procedures/interventions; 3) Morbid threats: *“do you want your baby to die*?*”*, coercion or extreme pressure from providers when clients declined interventions; 4) Compliance as valued: *“to be a ‘good client’”*, recounting compliance or obedience to medical staff recommendations as valuable social capital but suppressing desire to ask questions or decline care.

**Conclusion:**

We conclude that in situations where a pregnant person declines recommended treatment, or requests treatment that a care provider does not support, tension and strife may ensue. These situations deprioritize and decenter a woman’s autonomy and preferences, leading care providers and the culture of care away from the principles of respect and person-centred care.

## Introduction

In the past decade, respect during pregnancy and childbirth has emerged as one of the most valued aspects of care for service users worldwide [1–9]. A wealth of research documents the wants and needs of service users in the perinatal period that expand beyond physiologic outcomes to include psychosocial and moralistic aspects of care. As a result, the WHO set Standards for Quality Maternal Newborn Care in Health Facilities, and the Lancet recently published a Quality Maternal Newborn Care (QMNC) framework that both emphasize the centrality of the *experience* of maternity care [10]. The QMNC framework was developed by a multi-disciplinary expert stakeholder group who synthesized high-quality quantitative and qualitative evidence to recognize the values and philosophies held by service users, care providers, and health systems [10–12]. This comprehensive framework crystalizes the interdependence of various institutional, organizational, social, and interpersonal factors that interact to shape how quality maternity care can be realized.

Unfortunately, most North American healthcare systems continue to rely on a limited definition of quality, based on clinical metrics. This leads to dualistic thinking that values biomedical measures over psychosocial experiences and demonstrates the divide between what women value and what the system values [13, 14]. As human rights scholar Lynn Freedman writes, “The point is not that global strategies, evidence-based guidelines, or high-level monitoring and accountability initiatives are inherently wrong or unnecessary. But when they consume most of the oxygen in the room, drowning out voices and signals coming from the ground, they distort both understanding and action” [15, p.2069].

Thoughtful consideration of what maternity care users value includes not only what is lacking from one’s care, but also what is satisfying and generative. Across groups, core values of respect, thoughtful communication, personalized care, trust, and supporting one’s agency are consistently reported as integral to creating high-quality experiences for women [5, 7, 16]. Childbearing people desire agency and control over their bodies and care during pregnancy and birth and consider these rights as essential elements to achieving optimum health outcomes [7, 17, 18]. Other features include supportive patient-provider relationships, effective communication, demonstration of caring, and perceived competence [19–22]. Factors that impede high quality include disrespect, discrimination, non-consented care, abandonment, verbal abuse, physical abuse, and intimidation [23–26].

Poor treatment is a global phenomenon not confined to low and middle-income countries. In a large US-based study (n = 2700) conducted by Vedam, Stoll, Taiwo, et al., one in six women reported mistreatment at some point in their care, with verbal abuse (defined as shouting or scolding) most commonly reported [25]. In Canada, women reported rude and inappropriate behaviour by providers, lack of choice and informed consent, lack of respect when asserting one’s autonomy, and concerns about quality of care and attitudes toward marginalized and underserved populations [27].

For historically marginalized groups, mistreatment is linked to structures of racism, colonization, disenfranchisement, and chronic systemic oppression [28]. In the Giving Voice to Mothers study, investigators found that Black, Hispanic, Asian, and Indigenous women were twice as likely as White women to report that a healthcare provider ignored them, refused their request for help, or failed to respond to requests for help in a reasonable amount of time [25]. Research further confirms that Black and other women of colour cite factors such as fear, paternalistic care, power differentials, blatant discrimination, and active mistrust for maternal healthcare providers as having a harmful and marginalizing impact on their care [20, 29–31]. Marginalized groups in Canada, such as Indigenous and immigrant women, also described distressing experiences that include lack of choice, dismissive provider behaviours, and resource scarcity that contributed to feelings of having little to no power, choice, or control [32, 33].

At the core of many of these harmful experiences lie interactions with healthcare providers. In a meta-ethnographic review, Elmir et al. reviewed traumatic experiences of birth and noted shared and foreboding characteristics reported by those who had traumatic experiences during labour and/or birth [34]. Many felt they were not able to actively participate in decision-making about their own care and identified disturbing and belittling communications with healthcare professionals as definitive factors. Others felt betrayed by providers, who either actively ignored their opinions or did not invite their participatory communication [34, 35]. At the extreme, women also reported the dehumanizing experience of being objectified by their care providers, which manifested in being threatened, coerced, or even subjected to physical violence and abuse.

One aspect of disrespect and mistreatment is when patient autonomy feels tenuous or disregarded in situations where care is declined or refused. The ability to exercise autonomy through choice of care options depends on many factors, from an individual’s wants, needs, culture, and prior experiences to social, political, economic, and cultural attitudes and beliefs. [36, 37]. Analyses of how autonomy is facilitated in birth practices show that adherence to the principle of autonomy is not absolute but is stratified by race, gender, and provider type. [8, 14, 38, 39].

Examining experiences of declining recommended perinatal care can serve as a proxy to understand how autonomy is exercised. Jenkinson et al. reported that the pressure to accept unwanted care prevailed under the scepter of risk aversion [40]. A qualitative study by Ebert et al. found that participants felt insecure exercising their autonomous choice or seeking control in their care encounters [41]. They identified several barriers, including inadequate information, perceptions of heightened risks if they did not conform to routine procedures, and concerns about the actions and reactions of midwives when asserting personal choice. To explore the impacts on quality of care, including the experiences and perspective of service users who have declined aspects of their maternity care, we evaluated qualitative data provided from participants in the *Changing Childbirth in British Columbia (CCinBC)* Study that specifically asked about experiences of declining care.

## Methods

In 2014, *Changing Childbirth in BC* became the first provincial study to use a community-based participatory research (CBPR) approach to examine experiences of pregnancy and childbirth [8]. This approach gives authority to voices historically marginalized in research.

Women of childbearing age from different cultural and socio-economic backgrounds designed a mixed-methods study utilizing a cross-sectional survey. This paper offers a qualitative analysis of a large dataset of open-ended responses from a short answer items included in the online cross-sectional survey. A commitment to our community partners, in analyzing and disseminating findings, included reporting all of the data, including the narrative portions.

### Survey development

An online consultation with 1300 women of childbearing age determined key areas for study. A Steering Council, comprised of maternity service users, researchers, clinicians, and non-governmental organization (NGO) leaders then identified preferred modes of data collection and reviewed the literature to collect relevant, validated items for a cross-sectional survey. The team assessed each item for importance, relevance, clarity and designed new items for novel patient-oriented topics. The final instrument included information on preferences for and access to maternity care, maternal and newborn outcomes, and experiences of decision-making, respect, autonomy, and non-consented care [42]. Open-ended questions were used to ask participants to describe what aspects of care they declined, why they declined it, their perceptions of their provider’s reactions, and the resulting impact on their experiences.

### Recruitment

Following approval by the University of British Columbia Ethics Board (#H12-02418), all NGO and community partners disseminated a survey link and information about the study from January through June of 2014 through a public website to women of childbearing age across BC. Reminder notices were sent by email, postcards, community list-serves, NGO websites, and advertised through posters, and social media outlets. Participants engaged with the survey items after reviewing embedded formal consent language aligned with UBC Ethics guidelines. All institutional partner organizations, including a large provincial tertiary hospital and maternity clinics, recruited study participants and provided staff and space to support data collection. Details on methods for instrument development, data collection have been published previously [42, 43].

### Sample

Detailed information about the sociodemographics of the geographically representative total sample for the CCinBC study (n = 2051) are published elsewhere [8, 43]. Childbearing people who planned to have a baby, were expecting a baby or have had a baby in British Columbia at any time were eligible to participate. Minors were excluded. Respondents reported on events when they declined any aspect of their maternity care. "Care" is defined as “anything that might be done or given to either you or your baby, or that you were asked to do (take a test, treatment, medicine, etc.).” Among the subset of 1958 women who answered this question, 892 participants confirmed declining some aspects of their care, offering the 1540 narrative comments analyzed here.

### Analysis

We utilized a systematic and comprehensive approach to thematic analysis, as described by Braun & Clark, that includes immersion in the data, code development, and pattern recognition, leading to iterative generation of themes as a means to develop a rich description of the phenomenon of refusing care from the perspective of maternity service users [44]. According to Braun and Clarke, surveys with short answers can generate valid accounts of participants’ subjective experiences and narratives, making them a worthy data source for in-depth analysis [45]. We also The analysis team was intentionally interprofessional and included input from community members who served on the study Steering Council. Research team members analyzed the first 100 short answer comments to develop initial codes. After multiple iterations, one main coder finalized the codebook after seeking peer review and debriefing to build consensus throughout the coding process. See Table 1 for a sample portion of codes from the codebook. The analysis team also acknowledge their own positions as midwives, researchers, clinicians, and mothers who have themselves received maternity care reflecting varying types of experiences with pregnancy and childbirth. These included perceived experiences ranging from high-quality, respectful care to incidences of disconnect and tension with healthcare providers when refusing or declining procedures or clinical management. Responses ranged from short answers with a few words to one or more paragraphs. NVivo qualitative data analysis software supported data management and analysis.

**Table 1:** Themes and excerpted codes.

Theme	Sample Codes
Contentious interactions: *“I fought my entire way”*	• Mistrust
	• Disagreeing with care
	• Bullying by providers
Knowledge as control—Knowledge as power	• Being prepared with information
	• Clinical information
	• Embodied knowledge
Morbid threats: “*Do you want your baby to die*?”	• Manipulation
	• Threatening communication
	• Feeling scared
Compliance as valued: “*to be a "good client*."	• Being ‘good’
	• Obedience
	• Don’t ask questions

### A critical feminist approach

The promotion of bodily autonomy and full sovereignty in decision-making processes are both core concepts in feminist analysis of reproductive healthcare. Application of critical feminist theory to healthcare interactions offers an opportunity to examine concepts of domination and disrespect as tools of power within a system, as well as within patient-provider relationships [46]. As person-centred care becomes a foundation of equitable healthcare practice, growing concern over the misuse of medical power in obstetrics suggests that the application of this theoretical framework may offer insight into approaches to care that honour an individual’s sovereignty and personal or cultural choices. Clinical dominance in childbirth is considered a form of gender-based oppression [38]. We offer a feminist analysis that exposes the historical struggle to assert women’s rights in hegemonic professional spaces such as medicine. We examine and centralize questions about how systemic power is articulated in people’s lived experiences based on their identities, position in social hierarchies, and other forms of personal, political, and economic power [47]. Though our work is not primarily focused on race, we acknowledge the work of Black feminist theorists in laying the foundation to conceptualizing how intersectional factors are deeply embedded in the assertion of power [47–49].

## Findings

### Sample characteristics

The overall data set included 2100 childbearing who reported on 3586 maternity care experiences. If a participant reported that they “refused” or declined any aspect of their care, they were asked to further describe this experience using free text. Of the 1123 respondents, we counted 1540 responses, which means that some participants recounted experiences of more than one pregnancy. The majority of people who reported declining care (89.3%) experienced their last birth in British Columbia between 2010–2014 (i.e. within 5 years of data collection). Sociodemographic characteristics of those who reported declining care, compared to those who did not report declining care are reported in Table 2. The two groups are comparable on most characteristics with the exception of prenatal care provider and mode of birth. People who provided comments about declining care were more likely to receive care from midwives and less likely to give birth by Cesarean. Among *all* respondents, 7.3% self-identified as women of colour, 1.5% as First Nations, Inuit, or Métis, and 91.2% as white. Geographically, participants represented variation in health service areas of British Columbia: 702 (28.6%) responded from Vancouver Coastal, 717 (29.3%) from Fraser, 537 (21.9%) from Vancouver Island, 360 (14.7%) from the Interior and 135 (5.5%) from Northern Health Authority.

**Table 2:** Characteristics of childbearing people who declined care and provided open-ended comments (n = 2100).

	Declined care (n = 1123)	Did not decline care (n = 977)
Number of pregnancies (mean)	2.2	2.1
Number of children (mean)	1.7	1.6
Age at time of data collection (mean)	32.7	33.0
Identified with a vulnerable group*	90 (8.0)	70 (7.2)
Ethnicity		
Asian	26 (2.7)	32 (4.5)
White	875 (92.1)	655 (92.1)
Other	49 (5.2)	24 (3.4)
Missing	173	266
Highest level of education is Highschool	97 (10.1)	67 (9.4)
Family income $ 30,000 or less	75 (6.7)	47 (4.8)
Care provider during pregnancy		
Family Physician	225 (20.0)	313 (32.0)
Midwife	883 (78.6)	539 (55.2)
Obstetrician	208 (18.5)	259 (26.5)
Other	82	92
Multiple responses possible		
Gave birth by Cesarean section	184 (17.2)	212 (24.5)

*Identified as one or more of the following: family income < 30 k, immigrant or refugee, history of incarceration or housing instability).

#### Themes

Through our analysis, we developed four themes. 1) Contentious interactions: *“I fought my entire way”* describes the quality of interactions as being fraught with tension and opposition. Many participants recounted stories of “fighting” for the kind of care they wanted or having to “fight” when they did not want a particular procedure or intervention. 2) Knowledge as control—Knowledge as power: “*like I was a dim girl*” focuses attention on the value of knowledge as a source of power both for providers, considered keepers of medical knowledge, and for clients, who felt buttressed if they were knowledgeable about the procedures or interventions they were declining. 3) Morbid threats: “*Do you want your baby to die*?” encompasses the acute threats made by providers when clients refused or declined interventions. These were particularly powerful during labour and were often experienced as extreme coercion or manipulation to accept the procedures being offered. Lastly, participants described 4) Compliance as valued: “*to be a "good client*." Many recounted the sense that compliance or obedience to clinical recommendations was a deeply valued social currency in their healthcare experience, as well as something that could keep their questions or desire to decline care muted or suppressed.

#### Contentious interactions: “I fought my entire way”

This theme captures the notion that if service users refuse or decline aspects of their care, contentious and difficult interactions can ensue. This tension can then reverberate through various aspects of their care. One participant who miscarried at 17 weeks wrote, to describe her interaction with hospital staff:

*I fought my entire way through the processes of being able to meet him*, *hold him and care for him for the few hours after his passing*. *I even had to fight to birth him*. *The only option offered to me was D&C*. *The entire experience was surrounded by feelings of utter confusion… I felt horribly alone*.

This description offers insight into the lack of support she received after facing the challenge of an unanticipated loss. Another participant describes having to fight for what she wanted, regarding obstetric interventions:

*I fought induction*. *I trusted my body and my naturopath to get myself as healthy as possible*, *and it worked*. *I did it my way—but man*, *I had to fight*. *The OB at another point told me not to eat prior to a fetal monitoring test that she wanted me to take in the morning*, *so that I could have a c-section; to keep my options open*. *She didn’t hear that a c-section was not an "option" for me* … *neither did the mainstream midwife—who felt that I should have been induced at 38 weeks and told me she wouldn’t have taken me on had she known* …

Many participants described feeling pressured to comply to clinical management, often citing the notion of giving into pressure where they were not consulted or included in care decisions. In these situations, though they may have initially contested the clinical decision, they were worn out from the fight. One woman shared:

*When I was 7cm my contractions spaced out* … *I was tired and wanted to rest… but the midwives wanted to transfer to the hospital for oxytocin*. *I refused for a couple of hours*. *The midwives kept saying*, *"Don’t you want to get this over with sooner*?*" Of course*, *I wanted to*, *but that did not seem like a good enough reason to get oxytocin* … *Eventually I just got tired of saying no*, *so I agreed*. *…What was frustrating was that once we got to the hospital there were a series of events which were totally unnecessary… Overall it was a somewhat traumatizing birth*. *I felt so much conflict instead of joy* … *For days after*, *every time I closed my eyes I would have flashbacks of being for forced to lay back and people all standing in front of me and me screaming but no one can hear me*.

For this participant, not having her wishes respected led to a cascade of defeat that resembles classic markers of post-traumatic stress disorder. This feeling of resisting and then giving in was due to the various forms of coercion or pressure imposed upon her by providers.

Participants also described situations in which care providers pressured them to accept interventions or tests they did not want, without providing a clear rationale of why the intervention was needed. One woman described being “ganged up on” by care providers in her most vulnerable moment, while labouring:

*I first refused an epidural because I did not want one*. *After my husband left for a walk*, *[the] OB and nurse ganged up on me to have it*. *I felt totally defeated and gave in*. *I first refused magnesium sulfate because the nurse would not tell me the pros and cons after I asked a number of times*. *I reluctantly agreed (with uninformed consent) as I just couldn’t be heard by these "caregivers" and just gave up myself completely… I felt bullied and uninformed as to what it would do to me or our child*.

Relenting after fighting was common throughout the comments, which, coupled with a lack of knowledge or “uninformed consent”, seemed to occur when participants felt the most isolated, misguided, and even abandoned in their care.

In contrast, when procedures or prenatal investigations were presented as options rather than mandatory requirements that required compliance, participants felt valued and respected.

One participant articulated feeling respected in care:

*everything was presented as options to be considered and decided upon*, *it didn’t feel like I was ’refusing to accept care’ when I said no to things*. *And I did say no to a number of things* … *like prenatal testing*, *eye gel for the newborn*, *some of the gestational diabetes guidelines I was given* … *but none of that felt like refusing care*, *it was just part of the care I received while pregnant*.

This participant’s comment offers a nuanced understanding of what it means to take an active role in care decision-making, wherein declining options is an integral part of the process, not a contentious or tense interaction that leaves one feeling guarded or vulnerable.

#### Knowledge as control; Knowledge as power: “Like i was a dim girl”

At times, women felt that care providers used knowledge to control or manipulate their decisions and commented on how their own knowledge empowered them to remain rooted in their decisions. For some people, refusal led to a sense of being judged as ignorant or ill-informed. One participant’s experience illustrates the fear arising from care provider comments and framing of risk:

*The hospital obstetrician insisted that I would likely end up having a cesarean because I’m overweight*, *the baby was "overdue*,*" and "judging by my size" the baby would be a very large baby*. *He insisted that I save myself a long labour and inevitable emergency c-section*, *and just go ahead and book a cesarean*. *I refused*, *and he responded to me like I was a dim girl shirking his wise*, *professional advice; therefore*, *"endangering my life and the life of my baby*.*" I was extremely scared*, *but proceeded with a (successful) vaginal delivery*.

Patients are often encouraged by their providers to educate themselves about their healthcare. This is particularly true in labour and birth, where women may design a birth plan to codify self-advocacy and self-determination during their care.^14^ However, women who clearly research their options may feel disrespected and judged as being unknowledgeable if they refuse what is considered “routine” care:

*I did a lot of research prior to my daughter’s birth to understand what I could and could not refuse*, *while still keeping my daughter and I safe… [They] den[ied] delayed cord clamping*, *skin to skin contact and my husband cutting the cord*. *After my daughter’s birth I refused the oxytocin shot*, *knowing that my body would produce its own given the chance to nurse my baby*. *I refused the routine IV fluids and asked to drink water to rehydrate myself*, *and I asked not to have stitches*. *At the same time I was given a shot of oxytocin*, *stitches and an IV… they treated me like I knew nothing* … *I felt like a third class citizen*, *disrespected*, *stupid and very scared*, *realizing that I had absolutely no say in what they were going to do to me*.

This participant, armed with information, reports specific requests being ignored and, completely deprived of her power, likens this disempowerment to that experienced by a “third class” citizen. She links her refusal as the root cause for receiving poor quality of care, as if the refusal itself inspired retaliation. Her knowledge acquisition about routine procedures was ignored and weaponized against her, making her an “unruly” patient.

In contrast, robust discussions and feeling respected formed the basis of empowering experiences for participants, despite their decisions not to partake in routine interventions:

*We discussed these topics*, *I asked questions and expressed my concerns*, *she addressed my concerns directly and then left the decision up to me*. *I felt she provided me with enough information to make my own decisions and that she trusted me to do so…my midwife never seemed nervous or afraid of my decisions*, *and never tried to intimidate or influence me by emphasizing or exaggerating risk factors*.

This participant’s experience may indicate how knowledge-sharing, and respect for decisions that may not follow standard protocols serve to empower service users in their healthcare decisions.

#### Morbid threats: “Do you want your baby to die?”

Some women described being aggressively coerced into accepting interventions they did not want, and a few described feeling threatened by care providers. Here we highlight the problematic encounters where morbid threats as manipulative tactics assert power and control. One woman described being intimidated with guilt that she was endangering the life of her child:

*I was lied to and manipulated in response with phrases like*, *"Do you want your baby to die*? *Your baby will die if you don’t do this" even while the staff had no knowledge of the fetus’ condition other than its presentation*, *and aside of that they had no reason to suspect anything was amiss… When presented the [cesarean] consent form*, *my hand was held with the pen in it by a staff member until I was forced to make a mark—all the while [I was] saying that I wanted a vaginal birth and did not consent to a cesarean—then I was taken into the [operating room] and gassed*.

Episodes like this describe a communication technique used to coerce a decision, not to communicate concern or discuss implications.

The explicit assertion underlying these interactions is that a mother’s decision not to accept care recommendations was putting their infant’s life at risk. In the situation described below, a mother, wanting to breastfeed, declined formula. In response, she felt demoralized, unsupported, and worried that her ability to safely parent her child was being questioned.

*My baby had lost 11% of her body weight after 4 days (after birth) and they said I had to feed her formula* … *I had been struggling since birth with breastfeeding and felt completely unsupported… I said no to the formula and asked for a pump*. *They acted annoyed*, *shocked*, *and concerned by my refusal and kept warning me that pumping is a lot of work and continued to not offer assistance with breastfeeding… I also asked what would happen if we asked to be discharged*, *despite their recommendation*. *They implied that we would not receive the same quality of care… They also implied that my ability to protect my baby would be questioned*.

This theme reflects care provider reactions that assume decisions made in conflict with provider suggestions are morally questionable. Language that suggests death, endangerment, or negligence in communicating with patients was recounted in detail by some participants. Given the extreme nature of these accounts, communication appears to be weaponized and perceived as manipulative, and limiting to one’s autonomy.

#### Compliance as valued: “To be a ‘good client‴

Being compliant or adhering to care management was described as one way that women must negotiate the system’s demands. One woman described consenting to an investigation to please her providers and out of concern that declining care might impact her relationship with her midwives:

*These midwives requested that I have a second ultrasound at 38 weeks to ensure the baby was in the right position and that her size didn’t pose a risk for homebirth*. *I didn’t feel this test was necessary at all and I consented to it purely to humour my midwives*. *Because they were clearly uncomfortable with my refusal of some other tests*, *I felt I should be "reasonable" and consent to the ultrasound*. *As I was nearing my due date*, *I felt the need to be on good terms with my caregivers*. *The only reason I consented to this ultrasound was to be a "good client*.*"*

One participant described the significant harm triggered by care providers’ disregard for her previous history of trauma and use of guilt to manipulate her decision-making:

*I refused to labour on my back with my knees pinned to my chest*. *My midwife and a new nurse that came on shift… refused to accept me declining that position*. *For an hour they badgered me into it*, *even though I told my midwife that I had previously been raped*. *It felt like I was raped all over again*. *My midwife and the nurse kept asking me why I wouldn’t do "what was best for baby"*. *My midwife clearly acted like I was a terrible mother and a horrible person… I suffered from severe PTSD for 5 months afterwards*.

When procedures or interventions were presented instead as options rather than mandatory, patients felt empowered through the shared decision-making process:

*I declined a number of things during this pregnancy that I felt were unnecessary for me*. *My midwife talked me through everything*. *What the tests were for*, *alternatives*, *risks and benefits*. *She would tell me about them weeks ahead to give me time to research and make choices*. *Nothing mandatory was sprung on me at anytime*. *I felt educated*, *informed and confident when I made my choices*. *I could ask her opinion or recommendation and never feel like she was pushing me in any way*.

When declining requests or management decisions, participants reported that disrespect or disregard of their wishes led to an overall sense of mistrust that damaged the care alliance, as one experience illustrates:

*I had been very upset during my first pregnancy when I had a hospital birth*, *and several complete strangers came in and examined me*, *inserting their fingers into my vagina without even asking for consent… When I next showed up for an appointment*, *there was a practicum student waiting in the room*. *I respectfully reminded the midwife that I had chosen not to have a practicum student involved*. *The midwife said that she was here now*, *so couldn’t she just stay*. *I said sorry*, *but no… The practicum student left*, *but the midwife was visibly displeased*. *I felt stressed by this incident*, *and it negatively impacted my trust and respect for the midwife*.

Following advice, doing as one is told, and submitting to the status quo is a value that is perpetuated and demanded by the system. Providers often refer to patients being non-compliant or non-adherent to protocol as an interpersonal barrier, an attitude which is detrimental to the care alliance.

## Discussion

The *Changing Childbirth in BC* project was the first study in Canada to explore the experience of declining care offered or recommended by midwives, doctors, obstetricians, nurses, and other healthcare providers from the perspective of service users. This qualitative analysis deepens our understanding of factors that contribute to levels of autonomy and respect that were described by participants in the quantitative phase of the study [8]. The overall analysis shows a wide spectrum of how, why, and when care is declined, contested, or clearly refused. Some participants reported their bodily integrity being breached, such as by physical constraints placed on their movement or position during labour. Others reported cognitive constraints that limited their autonomy, choices, and care preferences, such as not supporting breastfeeding or strongly urging medicalized interventions (e.g. inductions or cesareans), leading to moral judgements and morbid threats. Due to various forms of pressure imposed by providers, participants felt vulnerable and ultimately relented to the interventions that they initially declined. Participants reported being unable to sustain the energy to continue declining given their position in the power dynamics of healthcare.

### Gendered systems of control

A feminist analysis of the experiences of pregnant and parenting people declining care demonstrates persistent gender-based power imbalances and tension between the assertion of autonomy against the rigidity of the medical model [50]. The medical model, rooted in a long history of control and authority over female bodies, remains the standard model of maternity care. The gendered power dynamics that define obstetrics, a historically male-dominated profession caring for female clients, persists and remains the elephant in the birth room. Legal scholar Michele Goodwin suggests that the rise of fetal protectionism persistently outweighs the rights of women–most acutely for marginalized people [51]. This stance is a key factor perpetuating the sublimation of maternal self-determination and autonomy [52, 53]. Power differentials are also apparent among professional groups, such as between nurses and physicians and midwives and physicians [46, 54]. These gendered dynamics are controlled by how organizations, such as healthcare, are historically oriented around power and perspective of medical doctors who were predominantly male. Healthcare is one institution where male power and domination is institutionalized, which inherently makes women, unless in the position of physician, often unable to access authority, whether she is a patient, nurse, or midwife provider [54].

Through this feminist analysis, we suggest that institutional practice and policies that do not actively support informed choice, even when the person’s preference reflects the best available evidence, is intrinsically rooted in the gendered oppression that has long shaped medical practice. Cahill identified recurring issues of paternalistic and defensive practice when providing patients with informed choices, particularly around cesareans [55]. Clinical decision-making, when solely based on physiological indicators, rigid adherence to protocols, poor communication, and documentation, persistently fails to acknowledge persons’ views, feelings, and embodied knowledge of their own health [56, 57]. The American College of Obstetricians and Gynecologists (ACOG) recognizes the inherent power dynamics that are present in healthcare interactions. In a practice bulletin, it affirms the presence of a “historical imbalance of power in gender relations and in the physician-patient relationship, the constraints on individual choice posed by complex medical technology, and the intersection of gender bias with race and class bias in the attitudes and actions of individuals and institutions [58].” Despite this best practice guideline, autonomy in care remains an elusive ideal.

At the extreme, some of the experiences recounted by participants can be categorized as human rights violations and instances of gender-based violence (GBV). Taket & Crisp propose that GBV is a systemic issue that pervades institutions, including healthcare. They lament that most initiatives seek to minimize the impact of GBV rather than prevent GBV, focusing on interpersonal interactions and eclipsing structural solutions [59]. Rights-based or person-centered care practices, including shared decision-making, are described as powerful tools to prevent GBV in healthcare settings [38]. As such, strategies to promote systemic access to respectful maternity care can contribute to the elimination of gender-based violence.

### Autonomy and quality of care

Loss of autonomy is frequently linked with negative or traumatic birth experiences [34, 56, 60] Stramrood et al. found that over a third of participants who recounted the circumstances of traumatic births attributed their trauma to “lack and/or loss of control, lack of communication/explanation, disrespect and mistreatment in care and the lack of emotional and/or practical support from caregivers” [61]. If respectful care is at the core of high-quality maternity care, understanding the circumstances of care where people feel minimized, ignored, disregarded, disrespected, or harmed becomes essential to building prevention strategies [62].

Contemporary researchers confirm a high prevalence of mistreatment in maternity care throughout the globe and include loss of autonomy and disrespect as core indicators of poor care quality. Bohren and colleagues provide ample evidence of mistreatment in low- and middle-income countries where global attention and advocacy have long been directed [24, 63] Meanwhile, research in high-income countries is also generating evidence that mistreatment and disrespect are a common experience in care [25, 29, 30]. Vedam, et al. noted that one in six women in the US report mistreatment during childbirth, confirming the pervasive nature of disrespect in care, including coercive and dismissive interactions with healthcare providers [25]. For women of colour, one in three reported mistreatment in their care. Notably, this rate for marginalized women in high-resource settings mirrors that reported by Bohren and colleagues in low-resource countries [64]. In childbearing, it appears that autonomy is not an absolute guarantee, leaving the pregnant person subject to the professional and cultural norms of the maternity care provider, unit, or system.

In the most recent *Health Care in Canada* survey, the highest rated priorities for service users were providers’ demonstration of a respectful and caring manner and making decisions in partnership with their providers [65]. Participants in the Changing Childbirth in BC study offered accounts of choosing to forego fetal testing with little resistance from their provider, to more acute scenarios where they were physically restrained during labour. Use of the term “refusal” posits service users as being in confrontation with the medical system, even when simply practicing their right to self-determination or advocating for their personal or cultural preferences. When care recommendations are declined, autonomy is actively asserted and, for the provider, medical power is directly challenged. These interactions are particularly strained during labour and birth, as the stakes appear to be higher. Prior scholarship shows that when perceptions of risk differ between providers and service users, a patient’s values, needs, and wishes are more likely to be sublimated [66, 67]. In a systematic review of person-centered care in birth facilities by Rubashkin et. al, five core objects emerged: autonomy, supportive care, social support, care environment, and dignity. Rubashkin and colleagues also suggest that challenges to autonomy are most often encountered during labour and birth [68]. Jenkinson et al. assert that declining care must be considered within a system defined to uphold “medical dominance and the patriarchal institution of motherhood”, with any deviation causing disruption to a system not built for flexibility and spontaneity [69].

Our study confirms that the experience of quality asserts itself through the actions, behaviors, and communication of providers, regardless of provider type. This is problematic, since midwifery claims a person-centered approach to perinatal care, with roots in a long tradition of resistance from medical dominance over childbearing [70]. Though midwifery ethic has long held the banner on the value of autonomy and decision-making, midwives in this study were not exempt from acting as agents of the medical system–a system that can unequivocally subsume the midwifery model of care [71].

### Implications for practice: Person-centered care

The widely used term “patient-centered care” (PCC) is used to describe an ideal vision of care wherein the patient’s wants, needs, opinions, and experiences are integrated as opposed to a system of care that is more “technology centred, doctor centred, hospital centred, disease centred” [72]. Liberati and colleagues describe PCC as materializing this rhetoric beyond the theoretical by attending to the patient-provider dyad in its interactions, communications, dignity, and empathic care [73].

Supporting patient autonomy requires bringing patient values into care planning, not as an event but as an ongoing process. Several participants recounted instances where they felt their decisions, even when contrary to their provider’s suggestion (or the standard of care), were respectfully accepted and honoured, offering a roadmap to approaches that encourage active participation in decision-making. The tendency of clinicians to expect and, at times, demand, patient “compliance” loses sight of the needs and circumstances of each individual person. Compliance as an unspoken social contract leads to women acquiescing and relinquishing parts of their autonomy and agency. Attanasio and Hardeman recently reported that, for Black and other women of colour, being “non-compliant” led to further mistreatment [74].

For person-centered care to be realized and activated, clinical guidelines and health policy, health advocacy, and patient education must help clients to practice autonomy and help providers to support autonomy [75]. In our study, interactions where women felt most unseen and unsupported were when their assertion of autonomy over their pregnancy or birth was ignored or denied. A core principle of PCC is that individual self-determination is not only tolerable but desirable, as it actively invites a dynamic of engagement that supports clients’ agency, self-determination, and body sovereignty [76].

In Canada, the patient bill of rights in the province of BC (known as the Health Care Consent Act) states that every adult has “the right to give consent or to refuse consent on any grounds, including moral or religious grounds, even if the refusal will result in death” [77]. It explicitly states that the healthcare system’s responsibility is to create a culture where individual providers are driven to engage in a person-centered decision-making process. Our research shows that this ethic of care is not universally accessible. Decisions are made within a complex interplay of values, preferences, experiences, and relationships [56]. Equitable and respectful care requires a paradigm shift in how clinicians view patients. Supporting a client’s autonomy, equality, and self-determination is essential in relational approaches to care and to realize the true values of person-centered care.

### Limitations

The findings of this study reflect childbearing people’s experiences of declining maternity care recommendations in British Columbia. The experiences of pregnant people may differ in other provinces or countries where social, institutional, or regulatory practices vary. Study participants were a self-selected group of service users who reported on declining aspects of care, and the sample may have been less representative of the experiences of the general population. We also note that this paper is based on a subsample of those who responded to the short answer questions–hence it we were not able to demonstrate whether this is a representative sample. This qualitative analysis captures only those comfortable writing in English and is predominantly composed of people who identified as white. While the Changing Childbirth in BC study was conducted in 2014, and currency of the findings may be of question, there are recent reports and evidence that mistreatment in health care, especially for marginalized populations, remains a persistent and relevant issue in healthcare quality.

## Conclusion

In situations where a pregnant person declines recommended treatment or requests treatment that a care provider does not support, tension and strife may ensue. These situations deprioritize and decenter a person’s autonomy and preferences, leading care providers and the culture of care away from the principles of respect and human rights. Our analysis of a large qualitative dataset elicits the complex characteristics of patient experiences of declining recommendations in a traditional, gendered healthcare system.

Deficits in autonomy and respect contribute to the overall quality of care and may have long-term adverse impacts on self-efficacy, mental health, and engagement with healthcare. If care is accepted as a relational dynamic, understanding how power is distributed in the maternity care relationship offers a critical perspective on considering declining care as a positive act of autonomy and self-determination, rather than one of incompetence, resistance, or disobedience.

## Supporting information

S1 File Changing childbirth in British Columbia–online survey PDF. (PDF)
