# Supplementary material for: “I fought my entire way”: Experiences of declining maternity care services in British Columbia
Source: PLoS One. 2021 Jun 4;16(6):e0252645. doi: 10.1371/journal.pone.0252645 (PMC8177419; doi:10.1371/journal.pone.0252645)
Supplement: S1 File — (PDF) [file pone.0252645.s001.pdf]

Researchers from the University of British Columbia invite you to participate in a Vancouver Foundation funded study about women's preferences and experiences with maternity care in BC.

To help us link you to the correct survey version, please choose the group you most identify with:

- I am a woman of childbearing age
- I am a woman of childbearing age who came to Canada within the last 5 years as an immigrant or refugee
- I am a woman of childbearing age who has been incarcerated
- I am a woman of childbearing age who is currently experiencing (or has in the past experienced) homelessness, poverty and/or other barriers
- I have a family member (e.g. partner, sister, daughter) who is expecting a baby or planning to become pregnant in BC
- None of the above

Welcome and thank you for your interest in our study!

<u>Changing Childbirth in BC:</u>
<u>Women working together to improve access to high quality maternity care</u>

This online survey will take about 20-30 minutes to complete if you have no children, but longer if you do have children. If you want to complete the survey in more than one session you can save it and continue later. Any information you give us as part of this survey will be kept strictly confidential.
Purpose: In this study we are looking at what might affect your experience of maternity care during pregnancy, birth and after birth. We want to understand how women and their families might benefit from being offered different types of care. This study also seeks to understand what women know about maternity care options, and what role they and their families play in making decisions during their pregnancy, labour and post-partum care. The results from this research will show us how to improve access to high quality maternity services for families in BC. You are being invited to take part in this study because you plan to have a baby, are currently expecting a baby or have had a baby in British Columbia.

Contact for information about the study:
If you have any questions or want more information about this study, please contact
Sunya Lai Thom, Project Coordinator, at communications@bcmidwives.com or 604 736 5976.

Technical Difficulties:
If you encounter any technical difficulties with the survey, please contact Kathrin Stoll at kstoll@alumni.ubc.ca

Informed Consent

Changing Childbirth in BC: Women working together to improve access to high quality maternity care

Principal Investigator: Professor Saraswathi Vedam
Department of Family Practice, Faculty of Medicine
University of British Columbia
Email: vedam@midwifery.ubc.ca

Co-Investigators: Ganga Jolicoeur
Executive Director
Midwives Association of British Columbia
Email: execdir@bcmidwives.com
604-736-5976
Kathrin Stoll
Postdoctoral Fellow, School of Population & Public Health, Faculty of Medicine
University of British Columbia
604-827-3305

Consent: Your participation in this study is entirely voluntary. You do not have to take part and you can decide to leave the study at any time. By filling out this survey, you consent to participate in the study.

Procedures: Supported by the UBC Division of Midwifery, the BC Women's Foundation, the Women's Health Research Institute and the Midwives Association of BC, women and their families will work with researchers to study access to high quality maternity care in several BC communities. Our team is interested in working with you to answer the following questions: What are women and their families' experiences of maternity care in BC? What aspects of maternity care are most important to women and their families? How do women and their partners choose a maternity care provider? Finally, what do women know about midwives in Canada?

Confidentiality: Only the lead researcher and her staff will ever see the private information that you share with us. Your name and any other information that identifies you personally will be kept private from anyone else. Answers to the questions will be separated from your personal information before they are studied. Your private information such as your name will also not be shown in any reports. In the results we will only use statistics, for example "10% of participants felt that…", and possibly some quotes that do not show any private information.

All files associated with this study, including your answers to the questions below will be stored on password protected computers. Online surveys will be hosted on secure Canadian servers with encryption features for added protection of data. All files (including print, online and electronic) will be destroyed 5 years after the study has come to an end.

Potential risks and benefits: We do not think there is anything in this study that could harm you or be bad for you. Some of the questions we ask might upset you although we do not expect them to. Your participation in this study will help researchers, maternity care providers, policy makers and health agencies understand what women and their families are looking for in terms of maternity care and how we can improve access to high quality maternity care for everyone.

If you have any concerns about your treatment or rights as a participant, you may contact the Research Participant Complaint Line in the University of British Columbia Office of Research Ethics by e-mail at RSIL@ors.ubc.ca or by phone at 604-822-8598 (Toll Free: 1-877-822-8598).

1. Have you ever given birth?

- Yes
- No
- No, I had a miscarriage or other loss during my pregnancy/pregnancies

2. Are you pregnant right now?

- Yes
- No

3. Have you been pregnant while living in British Columbia?

- Yes
- No

4. I live in BC and plan to become pregnant in the future

- Yes
- No
- Not sure
- N/A

5. I have a family member (e.g. partner, sister, daughter) who is expecting a baby or planning to become pregnant in BC

- Yes
- No
- N/A

5. How many times have you been pregnant before?

If you are pregnant now, please do not include this current pregnancy.

- 0
- 1
- 2
- 3
- 4
- 5
- 6
- 7
- 8
- 9
- 10
-

6. How many children do you have?

- 0
- 1
- 2
- 3
- 4
- 5
- 6
- 7
- 8
- 9
- 10

7. What was the most recent year that you gave birth?

____

8. Starting with your oldest child, when and where were your children born?

Child 1

Year of birth:	______________________
Country born in:	______________________
Province/State born in:	______________________

Child 2

Year of birth:	______________________
Country born in:	______________________
Province/State born in:	______________________

Child 3

Year of birth:	______________________
Country born in:	______________________
Province/State born in:	______________________

Child 4

Year of birth: ______________________

Country born in: ______________________

Province/State born in: ______________________

Child 5

Year of birth: ______________________

Country born in: ______________________

Province/State born in: ______________________

Child 6

Year of birth: ______________________

Country born in: ______________________

Province/State born in: ______________________

Child 7

Year of birth: ______________________

Country born in: ______________________

Province/State born in: ______________________

Child 8

Year of birth: ______________________

Country born in: ______________________

Province/State born in: ______________________

Child 9

Year of birth: ______________________

Country born in: ______________________

Province/State born in: ______________________

Child 10

Year of birth: ______________________

Country born in: ______________________

Province/State born in: ______________________

What Matters

1. When I was choosing my prenatal care provider I considered what I learned from...

Check all that apply:
- My/my partner's previous birth experiences
- My parent(s)
- Other family members
- Friends
- Co-workers
- Online connections (websites, parenting forum members)
- Community Health Service
- My family doctor
- Books about pregnancy and birth
- Settlement services
- Other ________________________

2. Who usually makes decisions about pregnancy, birth, and infant care in your family? (For example: choice of care provider, types of prenatal tests, birth location, etc.)

Check all that apply:
- I do
- My partner takes the lead
- My partner and I make the decision together
- My parents/elders lead the decisions (please specify: father, mother, father or mother in law) ________________________
- My partner and I make the decisions together with our elders ________________________
- Other, please explain: ________________________

3. Below are factors some women consider when choosing a maternity care provider. Please tell us how much of a factor each was in your choice of the maternity care provider for your recent prenatal care.

The maternity care provider...

	Major factor	Minor factor	Not a factor
Provided my prenatal care in a previous pregnancy	○	○	○
Had provided my well-woman (gyn) care	○	○	○
Was recommended by a health professional	○	○	○
Is highly rated on websites with information about specific care providers	○	○	○
Was a good match for what I value and want	○	○	○
Attends births at a hospital I like	○	○	○
Is female/included female providers	○	○	○
Was assigned to me as my maternity care provider	○	○	○

4. What is most important to you for your maternity and newborn care?

	Very Important	Important	Somewhat Important	Not Important
Choice of birthplace (home or hospital)	○	○	○	○
Having only one provider care for me	○	○	○	○
Having no more than 4 providers care for me	○	○	○	○

I lead the decisions about my pregnancy, birth and baby care				
My doctor or midwife guides the decisions				
Having support people of my choice present for labour and birth				
Having a provider who has expertise with natural methods for pain relief				
Having a provider who has expertise with high-risk pregnancies				
Having access to medicines for pain relief				
Knowing the doctor/midwife who will care for me during my birth				
Not being separated from my baby after birth				
Being able to choose a planned caesarean				
Having a pain-free birth				
Being cared for by my own family doctor				
Staying in my community for pregnancy and birth				
Having a provider who will do newborn care/breastfeeding support at my home				
Having enough time to ask questions and discuss my options				
Having a trusting relationship with my care provider				
Having a care provider who speaks my language				

Other (optional) ____________________

Other (optional) ____________________

Other (optional) ____________________

5. For any future births, how open would you be to giving birth...

	Definitely would not want this	Would consider this	Definitely would want this	I don't want to have any more biological children	Not sure
In a birth centre that is separate from a hospital					
At home					
In a hospital					

What Matters

1. If I get pregnant, I plan to seek maternity care from a:

- [ ] Midwife
- [ ] Family Doctor
- [ ] Obstetrician
- [ ] Health Centre Nurse
- [ ] No Provider (Please tell us why) ________________________

2. When I choose a prenatal care provider I will consider what I learn from...

Check all that apply:

- [ ] My partner
- [ ] My parent(s)
- [ ] Other family members
- [ ] Friends
- [ ] Co-workers
- [ ] Online connections (websites, parenting forum members)
- [ ] Community Health Service
- [ ] My family doctor
- [ ] Books about pregnancy and birth
- [ ] Settlement services
- [ ] Other ________________________

3. Who would make the decisions about pregnancy, birth, and infant care in your family? (For example: choice of care provider, types of prenatal tests, birth location, etc.)

Check all that apply:

- [ ] I would
- [ ] My partner would take the lead
- [ ] My partner and I would make the decision together
- [ ] My parents/elders would lead the decisions (please specify: father, mother, father or mother in law) ________________________
- [ ] My partner and I would make the decisions together with our elders ________________________
- [ ] Other, please explain: ________________________

4. Below are factors some women consider when choosing a maternity care provider. Please tell us how much of a factor each would be in your choice of a maternity care provider.

The maternity care provider...

	Major factor	Minor factor	Not a factor
Provided my prenatal care in a previous pregnancy	○	○	○
Had provided my well-woman (gyn) care	○	○	○
Was recommended by a health professional	○	○	○
Is highly rated on websites with information about specific care providers	○	○	○
Was a good match for what I value and want	○	○	○
Attends births at a hospital I like	○	○	○
Is female/included female providers	○	○	○
Was assigned to me as my maternity care provider	○	○	○

5. What is most important to you for your maternity and newborn care?

	Very Important	Important	Somewhat Important	Not Important
Choice of birthplace (home or hospital)	○	○	○	○
Having only one provider care for me	○	○	○	○
Having no more than 4 providers care for me	○	○	○	○
I lead the decisions about my pregnancy, birth and baby care	○	○	○	○
My doctor or midwife guides the decisions	○	○	○	○
Having support people of my choice present for labour and birth	○	○	○	○
Having a provider who has expertise with natural methods for pain relief	○	○	○	○
Having a provider who has expertise with high-risk pregnancies	○	○	○	○
Having access to medicines for pain relief	○	○	○	○
Knowing the doctor/midwife who will care for me during my birth	○	○	○	○
Not being separated from my baby after birth	○	○	○	○
Being able to choose a planned caesarean	○	○	○	○
Having a pain-free birth	○	○	○	○
Being cared for by my own family doctor	○	○	○	○
Staying in my community for pregnancy and birth	○	○	○	○
Having a provider who will do newborn care/breastfeeding support at my home	○	○	○	○
Having enough time to ask questions and discuss my options	○	○	○	○
Having a trusting relationship with my care provider	○	○	○	○
Having a care provider who speaks my language	○	○	○	○

Other (optional) ____________________

Other (optional) ____________________

Other (optional) ____________________

6. How open would you be to giving birth...

	Definitely would not want this	Would consider this	Definitely would want this	I don't want to have any biological children	Not sure
In a birth centre that is separate from a hospital	○	○	○	○	○
At home	○	○	○	○	○

In a hospital	○	○	○	○	○

Pregnancy History: Pregnancy 1

The next two sections collect information about how your maternity care either met your needs and/or did not meet your needs and expectations. Please choose one or two of your pregnancy or birth experiences to describe. You will be able to describe your experiences with more than one care provider for the same pregnancy.

If you had a pregnancy that resulted in a miscarriage, stillbirth or neonatal loss, please select one of the following:

- ( ) d. I do not want to share any of my experience with this pregnancy
- ( ) a. I would like to answer ALL questions about this pregnancy
- ( ) c. I would like to answer questions about pregnancy, labour and birth ONLY during this pregnancy
- ( ) b. I would like to answer questions about decision making ONLY during this pregnancy

1. Who was your care provider during your pregnancy?

Please check all that apply. You will have an opportunity to tell us your experiences with up to 3 providers for one pregnancy later on in the survey.

- [ ] Family Doctor
- [ ] Obstetrician
- [ ] Midwife
- [ ] Health Centre Nurse
- [ ] None
- [ ] Other ________________________

If you had no care provider for this pregnancy please tell us why:

____
____
____

If you had more than one main care provider during your pregnancy please indicate why:

Please check all the apply:

- [ ] Change of provider due to medical concerns
- [ ] Change of provider due to preference
- [ ] Change of provider due to limited maternity services in my community
- [ ] Other, please specify... ________________________

2. Were you expecting twins?

- ( ) Yes
- ( ) No

3. During this pregnancy I or my unborn babies had:

Please check all that apply:

- [ ] No health problems
- [ ] High blood pressure
- [ ] Depression
- [ ] Problems with the babies' growth
- [ ] Problems with the babies' health ________________________
- [ ] Diabetes
- [ ] Housing difficulties
- [ ] No support from friends or family
- [ ] Miscarriage
- [ ] Other ________________________

3. During this pregnancy I or my unborn baby had:

Please check all that apply:
- ☐ No health problems
- ☐ High blood pressure
- ☐ Depression
- ☐ Problems with baby's growth
- ☐ Problems with baby's health ________________________
- ☐ Diabetes
- ☐ Housing difficulties
- ☐ No support from friends or family
- ☐ Miscarriage
- ☐ Other ________________________

4. During labour, did you feel pressured from any health professional to have...

	Yes	No
Labour induction	○	○
An epidural	○	○
A cesarean	○	○

5. My labour was induced

- ○ Yes
- ○ No

Why was your labour induced?

Choose the reason that best applies to your situation:
- A care provider was concerned about the size of the baby
- A care provider was concerned that I was "overdue"
- My water had broken and there was fear of infection
- A care provider was concerned that the amniotic fluid around the baby was low
- A care provider was concerned that the baby was not doing well and needed to be born soon
- I had a health problem that required quick delivery of the baby
- I wanted to get the pregnancy over with
- I wanted to control the timing for work or other personal reasons
- I wanted to give birth with a specific provider
- Baby was full term, it was close to my due date
- Some other reason

6. I had a:

- ○ Spontaneous vaginal birth
- ○ Vaginal birth with forceps or vacuum
- ○ Caesarean birth

Whose idea was it for you to have a cesarean?

Please select the choice that best describes whose idea it was:
- ○ Mine, I decided I wanted the cesarean before I went into labour
- ○ Mine, I asked for the cesarean while I was in labour
- ○ My maternity care provider recommended a cesarean before I went into labour
- ○ My maternity care provider recommended a cesarean while I was in labour
- ○ Other ________________________

What was the reason for the cesarean?

Choose the reason that best applies to your situation:
- There was no medical reason
- I had had a prior cesarean
- Labour was taking too long

- Baby was in the wrong position
- The fetal monitor showed the baby was having problems during labour
- My maternity care provider worried that the baby was too big
- There was a problem with the placenta
- I had a health condition that called for this procedure
- I was past my due date
- My health care provider tried to induce my labour, but it didn't work
- I was afraid to go into labour and have my baby vaginally
- Baby was having trouble fitting through
- Some other reason

7. Where were your babies born?

Please select one of the following:

- Hospital, planned hospital birth
- Hospital, transfer from planned home birth
- Home, unplanned
- Home, planned
- Birth Centre
- Health Centre
- Other ________________________

7. Where was your baby born?

Please select one of the following:

- Hospital, planned hospital birth
- Hospital, transfer from planned home birth
- Home, unplanned
- Home, planned
- Birth Centre
- Health Centre
- Other ________________________

8. After labour, were you healthy and feeling well?

- Yes
- No

Comments (Optional):

____
____
____

9. Was your baby healthy and doing well?

- Yes
- No

Comments (Optional):

____
____
____

9. Were your babies healthy and doing well?

- Yes
- No

What kind of problems did your baby have?

Please select all the apply:

- Breathing problems

- Digestive track problems
- Fever or infection
- Jaundice
- Birth defect
- Low weight gain/"failure to thrive"
- Dehydration
- Stillbirth
- Neonatal Loss
- Congenital or genetic problem
- Other
- Decline to answer

What kind of problems did your babies have?

Please select all the apply:

- Breathing problems
- Digestive track problems
- Fever or infection
- Jaundice
- Birth defect
- Low weight gain/"failure to thrive"
- Dehydration
- Stillbirth
- Neonatal Loss
- Congenital or genetic problem
- Other
- Decline to answer

Comments (Optional):

____

____

____

10. As you came to the end of your pregnancy, how had you hoped to feed your baby?

- Breastfeeding only
- Formula only
- Both breast milk and formula

11. Two weeks after you gave birth, how were you feeding your baby?

- Breast milk only
- Formula only
- Both breast milk and formula

Decision Making: Pregnancy 1

Please describe your experiences when making decisions and choosing options for care during this pregnancy.

1. The following answers describe my conversations or experiences with my____________

Please select one:

- Family Doctor
- Obstetrician
- Midwife
- Health Centre Nurse
- Other _______________________

2. Decision making process:

Check the box that responds to your answer

	Completely Disagree	Strongly Disagree	Somewhat Disagree	Somewhat Agree	Strongly Agree	Completely Agree	N/A
My midwife asked me how involved in decision making I wanted to be	○	○	○	○	○	○	○
My midwife told me that there are different options for my maternity care	○	○	○	○	○	○	○
My midwife explained the advantages and disadvantages of the maternity care options	○	○	○	○	○	○	○
My midwife helped me understand all the information	○	○	○	○	○	○	○
I was given enough time to thoroughly consider the different care options	○	○	○	○	○	○	○
I was able to choose what I considered to be the best care options	○	○	○	○	○	○	○
My midwife respected that choice	○	○	○	○	○	○	○
I deferred to my midwife's advice	○	○	○	○	○	○	○

2. Decision making process:

Check the box that responds to your answer

	Completely Disagree	Strongly Disagree	Somewhat Disagree	Somewhat Agree	Strongly Agree	Completely Agree	N/A
My family doctor asked me how involved in decision making I wanted to be	○	○	○	○	○	○	○
My family doctor told me that there are different options for my maternity care	○	○	○	○	○	○	○

My family doctor explained the advantages and disadvantages of the maternity care options							
My family doctor helped me understand all the information							
I was given enough time to thoroughly consider the different care options							
I was able to choose what I considered to be the best care options							
My family doctor respected that choice							
I deferred to my family doctor's advice							

2. Decision making process:

Check the box that responds to your answer

	Completely Disagree	Strongly Disagree	Somewhat Disagree	Somewhat Agree	Strongly Agree	Completely Agree	N/A
My obstetrician asked me how involved in decision making I wanted to be							
My obstetrician told me that there are different options for my maternity care							
My obstetrician explained the advantages and disadvantages of the maternity care options							
My obstetrician helped me understand all the information							
I was given enough time to thoroughly consider the different care options							
I was able to choose what I considered to be the best care options							
My obstetrician respected that choice							
I deferred to my obstetrician's advice							

2. Decision making process:

Check the box that responds to your answer

	Completely Disagree	Strongly Disagree	Somewhat Disagree	Somewhat Agree	Strongly Agree	Completely Agree	N/A

My health centre nurse asked me how involved in decision making I wanted to be	○	○	○	○	○	○	○
My health centre nurse told me that there are different options for my maternity care	○	○	○	○	○	○	○
My health centre nurse explained the advantages and disadvantages of the maternity care options	○	○	○	○	○	○	○
My health centre nurse helped me understand all the information	○	○	○	○	○	○	○
I was given enough time to thoroughly consider the different care options	○	○	○	○	○	○	○
I was able to choose what I considered to be the best care options	○	○	○	○	○	○	○
My health centre nurse respected that choice	○	○	○	○	○	○	○
I deferred to my health centre nurse's advice	○	○	○	○	○	○	○

2. Decision making process:

Check the box that responds to your answer

	Completely Disagree	Strongly Disagree	Somewhat Disagree	Somewhat Agree	Strongly Agree	Completely Agree	N/A
My maternity care provider asked me how involved in decision making I wanted to be	○	○	○	○	○	○	○
My maternity care provider told me that there are different options for my maternity care	○	○	○	○	○	○	○
My maternity care provider explained the advantages and disadvantages of the maternity care options	○	○	○	○	○	○	○
My maternity care provider helped me understand all the information	○	○	○	○	○	○	○
I was given enough time to thoroughly consider the different care options	○	○	○	○	○	○	○
I was able to choose what I considered to be the best care options	○	○	○	○	○	○	○
My maternity care provider respected that choice	○	○	○	○	○	○	○

I deferred to my maternity care provider's advice	○	○	○	○	○	○	○

3. I was satisfied with my ability to participate in decision making for my...

Choose all that apply:

- ☐ Pregnancy
- ☐ Labour and birth
- ☐ After the birth
- ☐ Baby care
- ☐ None of the above
- ☐ N/A

4. Overall while making decisions during my pregnancy and birth care I felt:

	Yes	No	N/A
Comfortable asking questions	○	○	○
Comfortable declining care that was offered	○	○	○
Comfortable accepting the options for care that my midwife recommended	○	○	○
Coerced into accepting the options my midwife suggested	○	○	○
I chose the care options that I received	○	○	○
My personal preferences were respected	○	○	○
My cultural preferences were respected	○	○	○

4. Overall while making decisions during my pregnancy and birth care I felt:

	Yes	No	N/A
Comfortable asking questions	○	○	○
Comfortable declining care that was offered	○	○	○
Comfortable accepting the options for care that my family doctor recommended	○	○	○
Coerced into accepting the options my family doctor suggested	○	○	○
I chose the care options that I received	○	○	○
My personal preferences were respected	○	○	○
My cultural preferences were respected	○	○	○

4. Overall while making decisions during my pregnancy and birth care I felt:

	Yes	No	N/A
Comfortable asking questions	○	○	○
Comfortable declining care that was offered	○	○	○
Comfortable accepting the options for care that my obstetrician recommended	○	○	○
Coerced into accepting the options my obstetrician suggested	○	○	○
I chose the care options that I received	○	○	○
My personal preferences were respected	○	○	○

My cultural preferences were respected	○	○	○

4. Overall while making decisions during my pregnancy and birth care I felt:

	Yes	No	N/A
Comfortable asking questions	○	○	○
Comfortable declining care that was offered	○	○	○
Comfortable accepting the options for care that my health centre nurse recommended	○	○	○
Coerced into accepting the options my health centre nurse suggested	○	○	○
I chose the care options that I received	○	○	○
My personal preferences were respected	○	○	○
My cultural preferences were respected	○	○	○

4. Overall while making decisions during my pregnancy and birth care I felt:

	Yes	No	N/A
Comfortable asking questions	○	○	○
Comfortable declining care that was offered	○	○	○
Comfortable accepting the options for care that my maternity care provider recommended	○	○	○
Coerced into accepting the options my maternity care provider suggested	○	○	○
I chose the care options that I received	○	○	○
My personal preferences were respected	○	○	○
My cultural preferences were respected	○	○	○

Please add any additional comments:

Optional

____

5. At any time did you refuse to accept any care that a nurse, doctor or midwife offered to you or your baby? "Care" includes anything that might be done or given to either of you or that you were asked to do (take a test, treatment, medicine, etc.).

- ○ Yes
- ○ No
- ○ Decline to answer
- ○ N/A

Please tell us what you refused, why you refused it, how the staff reacted, and how you felt about it. We would appreciate as much detail as you would care to provide.

____
____
____

5. During this pregnancy, how many times did you expect to see your midwife before birth?

- ○ 4 or more
- ○ Less than 4 (Please explain) __________________________
- ○ Unsure
- ○ N/A

5. During this pregnancy, how many times did you expect to see your family doctor before birth?

- 4 or more
- Less than 4 (Please explain) ________________________
- Unsure
- N/A

5. During this pregnancy, how many times did you expect to see your obstetrician before birth?

- 4 or more
- Less than 4 (Please explain) ________________________
- Unsure
- N/A

5. During this pregnancy, how many times did you expect to see your health centre nurse before birth?

- 4 or more
- Less than 4 (Please explain) ________________________
- Unsure
- N/A

5. During this pregnancy, how many times did you expect to see your maternity care provider before birth?

- 4 or more
- Less than 4 (Please explain) ________________________
- Unsure
- N/A

6. How much time, on average, did you have at prenatal appointments?

- Less than 15 minutes
- 15-30 minutes
- 31-60 minutes
- More than 60 minutes
- N/A

7. I felt that time was:

- Too little
- Just enough
- Too much
- N/A

8. During a prenatal visit, did you ever hold back from asking questions or discussing your concerns because...

	No, never	Yes. once	Yes, more than once	N/A
Your maternity care provider seemed rushed				
You wanted maternity care that differed from what your maternity care provider recommended				
You thought your maternity care provider might think you were being difficult				

9. When you had your baby, how often were you treated poorly by your maternity care provider because of...

	Never	Sometimes	Usually	Always	N/A
Your race, ethnicity, cultural background or language					

Your sexual orientation and/or gender identity	◯	◯	◯	◯	◯
Your health insurance	◯	◯	◯	◯	◯
A difference in opinion with your caregivers about the right care for yourself or your baby	◯	◯	◯	◯	◯

Decision Making: Pregnancy 1 (Optional)

Please describe your experiences with your second care provider making decisions and choosing options for care during this pregnancy.

1. The following answers describe my conversations or experiences with my____________

Please select one:

- Family Doctor
- Obstetrician
- Midwife
- Health Centre Nurse
- Other ________________________

2. Decision making process:

Check the box that responds to your answer

	Completely Disagree	Strongly Disagree	Somewhat Disagree	Somewhat Agree	Strongly Agree	Completely Agree	N/A
My midwife asked me how involved in decision making I wanted to be							
My midwife told me that there are different options for my maternity care							
My midwife explained the advantages and disadvantages of the maternity care options							
My midwife helped me understand all the information							
I was given enough time to thoroughly consider the different care options							
I was able to choose what I considered to be the best care options							
My midwife respected that choice							
I deferred to my midwife's advice							

2. Decision making process:

Check the box that responds to your answer

	Completely Disagree	Strongly Disagree	Somewhat Disagree	Somewhat Agree	Strongly Agree	Completely Agree	N/A
My family doctor asked me how involved in decision making I wanted to be							

My family doctor told me that there are different options for my maternity care							
My family doctor explained the advantages and disadvantages of the maternity care options							
My family doctor helped me understand all the information							
I was given enough time to thoroughly consider the different care options							
I was able to choose what I considered to be the best care options							
My family doctor respected that choice							
I deferred to my family doctor's advice							

2. Decision making process:

Check the box that responds to your answer

	Completely Disagree	Strongly Disagree	Somewhat Disagree	Somewhat Agree	Strongly Agree	Completely Agree	N/A
My obstetrician asked me how involved in decision making I wanted to be							
My obstetrician told me that there are different options for my maternity care							
My obstetrician explained the advantages and disadvantages of the maternity care options							
My obstetrician helped me understand all the information							
I was given enough time to thoroughly consider the different care options							
I was able to choose what I considered to be the best care options							
My obstetrician respected that choice							
I deferred to my obstetrician's advice							

2. Decision making process:

Check the box that responds to your answer

	Completely Disagree	Strongly Disagree	Somewhat Disagree	Somewhat Agree	Strongly Agree	Completely Agree	N/A
My health centre nurse asked me how involved in decision making I wanted to be							
My health centre nurse told me that there are different options for my maternity care							
My health centre nurse explained the advantages and disadvantages of the maternity care options							
My health centre nurse helped me understand all the information							
I was given enough time to thoroughly consider the different care options							
I was able to choose what I considered to be the best care options							
My health centre nurse respected that choice							
I deferred to my health centre nurse's advice							

2. Decision making process:

Check the box that responds to your answer

	Completely Disagree	Strongly Disagree	Somewhat Disagree	Somewhat Agree	Strongly Agree	Completely Agree	N/A
My maternity care provider asked me how involved in decision making I wanted to be							
My maternity care provider told me that there are different options for my maternity care							
My maternity care provider explained the advantages and disadvantages of the maternity care options							
My maternity care provider helped me understand all the information							
I was given enough time to thoroughly consider the different care options							
I was able to choose what I considered to be the best care options							

My maternity care provider respected that choice	○	○	○	○	○	○	○
I deferred to my maternity care provider's advice	○	○	○	○	○	○	○

3. I was satisfied with my ability to participate in decision making for my...

Choose all that apply:

- ☐ Pregnancy
- ☐ Labour and birth
- ☐ After the birth
- ☐ Baby care
- ☐ None of the above

4. Overall while making decisions during my pregnancy and birth care I felt:

	Yes	No	N/A
Comfortable asking questions	○	○	○
Comfortable declining care that was offered	○	○	○
Comfortable accepting the options for care that my midwife recommended	○	○	○
Coerced into accepting the options my midwife suggested	○	○	○
I chose the care options that I received	○	○	○
My personal preferences were respected	○	○	○
My cultural preferences were respected	○	○	○

4. Overall while making decisions during my pregnancy and birth care I felt:

	Yes	No	N/A
Comfortable asking questions	○	○	○
Comfortable declining care that was offered	○	○	○
Comfortable accepting the options for care that my family doctor recommended	○	○	○
Coerced into accepting the options my family doctor suggested	○	○	○
I chose the care options that I received	○	○	○
My personal preferences were respected	○	○	○
My cultural preferences were respected	○	○	○

4. Overall while making decisions during my pregnancy and birth care I felt:

	Yes	No	N/A
Comfortable asking questions	○	○	○
Comfortable declining care that was offered	○	○	○
Comfortable accepting the options for care that my obstetrician recommended	○	○	○
Coerced into accepting the options my obstetrician suggested	○	○	○
I chose the care options that I received	○	○	○

My personal preferences were respected	◯	◯	◯
My cultural preferences were respected	◯	◯	◯

4. Overall while making decisions during my pregnancy and birth care I felt:

	Yes	No	N/A
Comfortable asking questions	◯	◯	◯
Comfortable declining care that was offered	◯	◯	◯
Comfortable accepting the options for care that my health centre nurse recommended	◯	◯	◯
Coerced into accepting the options my health centre nurse suggested	◯	◯	◯
I chose the care options that I received	◯	◯	◯
My personal preferences were respected	◯	◯	◯
My cultural preferences were respected	◯	◯	◯

4. Overall while making decisions during my pregnancy and birth care I felt:

	Yes	No	N/A
Comfortable asking questions	◯	◯	◯
Comfortable declining care that was offered	◯	◯	◯
Comfortable accepting the options for care that my maternity care provider recommended	◯	◯	◯
Coerced into accepting the options my maternity care provider suggested	◯	◯	◯
I chose the care options that I received	◯	◯	◯
My personal preferences were respected	◯	◯	◯
My cultural preferences were respected	◯	◯	◯

Please add any additional comments:

Optional

____

5. At any time did you refuse to accept any care that a nurse, doctor or midwife offered to you or your baby? "Care" includes anything that might be done or given to either of you or that you were asked to do (take a test, treatment, medicine, etc.).

◯ Yes
◯ No
◯ Decline to answer

Please tell us what you refused, why you refused it, how the staff reacted, and how you felt about it. We would appreciate as much detail as you would care to provide.

____
____
____

6. During this pregnancy, how many times do you expect to see your midwife before birth?

◯ 4 or more
◯ Less than 4 (Please explain) __________________________
◯ Unsure

6. During this pregnancy, how many times do you expect to see your family doctor before birth?

- 4 or more
- Less than 4 (Please explain) ________________________
- Unsure

6. During this pregnancy, how many times do you expect to see your obstetrician before birth?

- 4 or more
- Less than 4 (Please explain) ________________________
- Unsure

6. During this pregnancy, how many times do you expect to see your health centre nurse before birth?

- 4 or more
- Less than 4 (Please explain) ________________________
- Unsure

6. During this pregnancy, how many times do you expect to see your maternity care provider before birth?

- 4 or more
- Less than 4 (Please explain) ________________________
- Unsure

7. How much time, on average, did you have at prenatal appointments?

- Less than 15 minutes
- 15-30 minutes
- 31-60 minutes
- More than 60 minutes

8. I felt that time was:

- Too little
- Just enough
- Too much

9. During a prenatal visit, did you ever hold back from asking questions or discussing your concerns because...

	No, never	Yes, once	Yes, more than once
Your maternity care provider seemed rushed	◯	◯	◯
You wanted maternity care that differed from what your maternity care provider recommended	◯	◯	◯
You thought your maternity care provider might think you were being difficult	◯	◯	◯

10. When you had your baby, how often were you treated poorly by your maternity care provider because of...

	Never	Sometimes	Usually	Always
Your race, ethnicity, cultural background or language	◯	◯	◯	◯
Your sexual orientation and/or gender identity	◯	◯	◯	◯
Your health insurance	◯	◯	◯	◯
A difference in opinion with your caregivers about the right care for yourself or your baby	◯	◯	◯	◯

Decision Making: Pregnancy 1 (Optional)

Please describe your experiences with your third care provider making decisions and choosing options for care during this pregnancy.

1. The following answers describe my conversations or experiences with my____________

Please select one:
- Family Doctor
- Obstetrician
- Midwife
- Health Centre Nurse
- Other ________________________

2. Decision making process:

Check the box that responds to your answer

	Completely Disagree	Strongly Disagree	Somewhat Disagree	Somewhat Agree	Strongly Agree	Completely Agree	N/A
My midwife asked me how involved in decision making I wanted to be	○	○	○	○	○	○	○
My midwife told me that there are different options for my maternity care	○	○	○	○	○	○	○
My midwife explained the advantages and disadvantages of the maternity care options	○	○	○	○	○	○	○
My midwife helped me understand all the information	○	○	○	○	○	○	○
I was given enough time to thoroughly consider the different care options	○	○	○	○	○	○	○
I was able to choose what I considered to be the best care options	○	○	○	○	○	○	○
My midwife respected that choice	○	○	○	○	○	○	○
I deferred to my midwife's advice	○	○	○	○	○	○	○

2. Decision making process:

Check the box that responds to your answer

	Completely Disagree	Strongly Disagree	Somewhat Disagree	Somewhat Agree	Strongly Agree	Completely Agree	N/A
My family doctor asked me how involved in decision making I wanted to be	○	○	○	○	○	○	○

My family doctor told me that there are different options for my maternity care							
My family doctor explained the advantages and disadvantages of the maternity care options							
My family doctor helped me understand all the information							
I was given enough time to thoroughly consider the different care options							
I was able to choose what I considered to be the best care options							
My family doctor respected that choice							
I deferred to my family doctor's advice							

2. Decision making process:

Check the box that responds to your answer

	Completely Disagree	Strongly Disagree	Somewhat Disagree	Somewhat Agree	Strongly Agree	Completely Agree	N/A
My obstetrician asked me how involved in decision making I wanted to be							
My obstetrician told me that there are different options for my maternity care							
My obstetrician explained the advantages and disadvantages of the maternity care options							
My obstetrician helped me understand all the information							
I was given enough time to thoroughly consider the different care options							
I was able to choose what I considered to be the best care options							
My obstetrician respected that choice							
I deferred to my obstetrician's advice							

2. Decision making process:

Check the box that responds to your answer

	Completely Disagree	Strongly Disagree	Somewhat Disagree	Somewhat Agree	Strongly Agree	Completely Agree	N/A
My health centre nurse asked me how involved in decision making I wanted to be							
My health centre nurse told me that there are different options for my maternity care							
My health centre nurse explained the advantages and disadvantages of the maternity care options							
My health centre nurse helped me understand all the information							
I was given enough time to thoroughly consider the different care options							
I was able to choose what I considered to be the best care options							
My health centre nurse respected that choice							
I deferred to my health centre nurse's advice							

2. Decision making process:

Check the box that responds to your answer

	Completely Disagree	Strongly Disagree	Somewhat Disagree	Somewhat Agree	Strongly Agree	Completely Agree	N/A
My maternity care provider asked me how involved in decision making I wanted to be							
My maternity care provider told me that there are different options for my maternity care							
My maternity care provider explained the advantages and disadvantages of the maternity care options							
My maternity care provider helped me understand all the information							
I was given enough time to thoroughly consider the different care options							
I was able to choose what I considered to be the best care options							

My maternity care provider respected that choice	○	○	○	○	○	○	○
I deferred to my maternity care provider's advice	○	○	○	○	○	○	○

3. I was satisfied with my ability to participate in decision making for my...

Choose all that apply:

- ☐ Pregnancy
- ☐ Labour and birth
- ☐ After the birth
- ☐ Baby care
- ☐ None of the above

4. Overall while making decisions during my pregnancy and birth care I felt:

	Yes	No	N/A
Comfortable asking questions	○	○	○
Comfortable declining care that was offered	○	○	○
Comfortable accepting the options for care that my midwife recommended	○	○	○
Coerced into accepting the options my midwife suggested	○	○	○
I chose the care options that I received	○	○	○
My personal preferences were respected	○	○	○
My cultural preferences were respected	○	○	○

4. Overall while making decisions during my pregnancy and birth care I felt:

	Yes	No	N/A
Comfortable asking questions	○	○	○
Comfortable declining care that was offered	○	○	○
Comfortable accepting the options for care that my family doctor recommended	○	○	○
Coerced into accepting the options my family doctor suggested	○	○	○
I chose the care options that I received	○	○	○
My personal preferences were respected	○	○	○
My cultural preferences were respected	○	○	○

4. Overall while making decisions during my pregnancy and birth care I felt:

	Yes	No	N/A
Comfortable asking questions	○	○	○
Comfortable declining care that was offered	○	○	○
Comfortable accepting the options for care that my obstetrician recommended	○	○	○
Coerced into accepting the options my obstetrician suggested	○	○	○
I chose the care options that I received	○	○	○

My personal preferences were respected	○	○	○
My cultural preferences were respected	○	○	○

4. Overall while making decisions during my pregnancy and birth care I felt:

	Yes	No	N/A
Comfortable asking questions	○	○	○
Comfortable declining care that was offered	○	○	○
Comfortable accepting the options for care that my health centre nurse recommended	○	○	○
Coerced into accepting the options my health centre nurse suggested	○	○	○
I chose the care options that I received	○	○	○
My personal preferences were respected	○	○	○
My cultural preferences were respected	○	○	○

4. Overall while making decisions during my pregnancy and birth care I felt:

	Yes	No	N/A
Comfortable asking questions	○	○	○
Comfortable declining care that was offered	○	○	○
Comfortable accepting the options for care that my maternity care provider recommended	○	○	○
Coerced into accepting the options my maternity care provider suggested	○	○	○
I chose the care options that I received	○	○	○
My personal preferences were respected	○	○	○
My cultural preferences were respected	○	○	○

Please add any additional comments:

Optional

____

5. At any time did you refuse to accept any care that a nurse, doctor or midwife offered to you or your baby? "Care" includes anything that might be done or given to either of you or that you were asked to do (take a test, treatment, medicine, etc.).

○ Yes
○ No
○ Decline to answer

Please tell us what you refused, why you refused it, how the staff reacted, and how you felt about it. We would appreciate as much detail as you would care to provide.

____
____
____

6. During this pregnancy, how many times do you expect to see your midwife before birth?

○ 4 or more
○ Less than 4 (Please explain) __________________________
○ Unsure

6. During this pregnancy, how many times do you expect to see your family doctor before birth?

- 4 or more
- Less than 4 (Please explain) ________________________
- Unsure

6. During this pregnancy, how many times do you expect to see your obstetrician before birth?

- 4 or more
- Less than 4 (Please explain) ________________________
- Unsure

6. During this pregnancy, how many times do you expect to see your health centre nurse before birth?

- 4 or more
- Less than 4 (Please explain) ________________________
- Unsure

6. During this pregnancy, how many times do you expect to see your maternity care provider before birth?

- 4 or more
- Less than 4 (Please explain) ________________________
- Unsure

7. How much time, on average, did you have at prenatal appointments?

- Less than 15 minutes
- 15-30 minutes
- 31-60 minutes
- More than 60 minutes

8. I felt that time was:

- Too little
- Just enough
- Too much

9. During a prenatal visit, did you ever hold back from asking questions or discussing your concerns because...

	No, never	Yes, once	Yes, more than once
Your maternity care provider seemed rushed	○	○	○
You wanted maternity care that differed from what your maternity care provider recommended	○	○	○
You thought your maternity care provider might think you were being difficult	○	○	○

10. When you had your baby, how often were you treated poorly by your maternity care provider because of...

	Never	Sometimes	Usually	Always
Your race, ethnicity, cultural background or language	○	○	○	○
Your sexual orientation and/or gender identity	○	○	○	○
Your health insurance	○	○	○	○
A difference in opinion with your caregivers about the right care for yourself or your baby	○	○	○	○

Pregnancy History: Pregnancy 2

If you had a pregnancy that resulted in a miscarriage, stillbirth or neonatal loss, please select one of the following:

- d. I do not want to share my experience with this pregnancy
- a. I would like to answer ALL questions about this pregnancy
- c. I would like to answer questions about pregnancy, labour and birth ONLY during this pregnancy
- b. I would like to answer questions about decision making ONLY during this pregnancy

1. Who was your care provider during your pregnancy?

Please check all that apply. You will have an opportunity to tell us your experiences with up to 3 providers for one pregnancy later on in the survey.

- Family Doctor
- Obstetrician
- Midwife
- Health Centre Nurse
- None
- Other ________________________

If you had no care provider for this pregnancy please tell us why:

____
____
____

If you had more than one main care provider during your pregnancy please indicate why:

Please check all the apply:

- Change of provider due to medical concerns
- Change of provider due to preference
- Change of provider due to limited maternity services in my community
- Other, please specify... ________________________

2. Were you expecting twins?

- Yes
- No

3. During this pregnancy I or my unborn baby had:

Please check all that apply:

- No health problems
- High blood pressure
- Depression
- Problems with baby's growth
- Problems with baby's health ________________________
- Diabetes
- Housing difficulties
- No support from friends or family
- Miscarriage
- Other ________________________

3. During this pregnancy I or my unborn babies had:

Please check all that apply:

- No health problems
- High blood pressure
- Depression

- [ ] Problems with the babies' growth
- [ ] Problems with the babies' health ________________________
- [ ] Diabetes
- [ ] Housing difficulties
- [ ] No support from friends or family
- [ ] Miscarriage
- [ ] Other ________________________

4. During labour, did you feel pressured from any health professional to have...

	Yes	No
Labour induction	○	○
An epidural	○	○
A cesarean	○	○

5. I had a:

○ Spontaneous vaginal birth
○ Vaginal birth with forceps or vacuum
○ Caesarean birth

6. My labour was induced

○ Yes
○ No

Why was your labour induced?

Choose the reason that best applies to your situation:
- A care provider was concerned about the size of the baby
- A care provider was concerned that I was "overdue"
- My water had broken and there was fear of infection
- A care provider was concerned that the amniotic fluid around the baby was low
- A care provider was concerned that the baby was not doing well and needed to be born soon
- I had a health problem that required quick delivery of the baby
- I wanted to get the pregnancy over with
- I wanted to control the timing for work or other personal reasons
- I wanted to give birth with a specific provider
- Baby was full term, it was close to my due date
- Some other reason

Whose idea was it for you to have a cesarean?

Please select the choice that best describes whose idea it was:
○ Mine, I decided I wanted the cesarean before I went into labour
○ Mine, I asked for the cesarean while I was in labour
○ My maternity care provider recommended a cesarean before I went into labour
○ My maternity care provider recommended a cesarean while I was in labour
○ Other ________________________

What was the reason for the cesarean?

Choose the reason that best applies to your situation:
- There was no medical reason
- I had had a prior cesarean
- Labour was taking too long
- Baby was in the wrong position
- The fetal monitor showed the baby was having problems during labour
- My maternity care provider worried that the baby was too big
- There was a problem with the placenta

• I had a health condition that called for this procedure
• I was past my due date
• My health care provider tried to induce my labour, but it didn't work
• I was afraid to go into labour and have my baby vaginally
• Baby was having trouble fitting through
• Some other reason

7. Where was your baby born?

Please select one of the following:
☐ Hospital, planned hospital birth
☐ Hospital, transfer from planned home birth
☐ Home, unplanned
☐ Home, planned
☐ Birth Centre
☐ Health Centre
☐ Other ________________________

7. Where were your babies born?

Please select one of the following:
○ Hospital, planned hospital birth
○ Hospital, transfer from planned home birth
○ Home, unplanned
○ Home, planned
○ Birth Centre
○ Health Centre
○ Other ________________________

8. After labour, were you healthy and feeling well?

○ Yes
○ No

Comments (Optional):

____
____
____

9. Was your baby healthy and doing well?

○ Yes
○ No

Comments (Optional):

____
____
____

9. Were your babies healthy and doing well?

○ Yes
○ No

What kind of problems did your baby have?

Please select all the apply:
☐ Breathing problems
☐ Digestive track problems
☐ Fever or infection
☐ Jaundice
☐ Birth defect

- Low weight gain/"failure to thrive"
- Dehydration
- Stillbirth
- Neonatal loss
- Congenital or genetic problem
- Other
- Decline to answer

What kind of problems did your babies have?

Please select all the apply:

- Breathing problems
- Digestive track problems
- Fever or infection
- Jaundice
- Birth defect
- Low weight gain/"failure to thrive"
- Dehydration
- Stillbirth
- Neonatal loss
- Congenital or genetic problem
- Other
- Decline to answer

Comments (Optional):

____
____
____

10. As you came to the end of your pregnancy, how had you hoped to feed your baby?

- Breastfeeding only
- Formula only
- Both breast milk and formula

11. Two weeks after you gave birth, how were you feeding your baby?

- Breast milk only
- Formula only
- Both breast milk and formula

Decision Making: Pregnancy 2

Please describe your experiences when making decisions and choosing options for care during this pregnancy.

1. The following answers describe my conversations or experiences with my____________

Please select one:
- ◯ Family Doctor
- ◯ Obstetrician
- ◯ Midwife
- ◯ Health Centre Nurse
- ◯ Other ________________________

2. Decision making process:

Check the box that responds to your answer

	Completely Disagree	Strongly Disagree	Somewhat Disagree	Somewhat Agree	Strongly Agree	Completely Agree	N/A
My midwife asked me how involved in decision making I wanted to be	◯	◯	◯	◯	◯	◯	◯
My midwife told me that there are different options for my maternity care	◯	◯	◯	◯	◯	◯	◯
My midwife explained the advantages and disadvantages of the maternity care options	◯	◯	◯	◯	◯	◯	◯
My midwife helped me understand all the information	◯	◯	◯	◯	◯	◯	◯
I was given enough time to thoroughly consider the different care options	◯	◯	◯	◯	◯	◯	◯
I was able to choose what I considered to be the best care options	◯	◯	◯	◯	◯	◯	◯
My midwife respected that choice	◯	◯	◯	◯	◯	◯	◯
I deferred to my midwife's advice	◯	◯	◯	◯	◯	◯	◯

2. Decision making process:

Check the box that responds to your answer

	Completely Disagree	Strongly Disagree	Somewhat Disagree	Somewhat Agree	Strongly Agree	Completely Agree	N/A
My family doctor asked me how involved in decision making I wanted to be	◯	◯	◯	◯	◯	◯	◯
My family doctor told me that there are different options for my maternity care	◯	◯	◯	◯	◯	◯	◯

My family doctor explained the advantages and disadvantages of the maternity care options	○	○	○	○	○	○	○
My family doctor helped me understand all the information	○	○	○	○	○	○	○
I was given enough time to thoroughly consider the different care options	○	○	○	○	○	○	○
I was able to choose what I considered to be the best care options	○	○	○	○	○	○	○
My family doctor respected that choice	○	○	○	○	○	○	○
I deferred to my family doctor's advice	○	○	○	○	○	○	○

2. Decision making process:

Check the box that responds to your answer

	Completely Disagree	Strongly Disagree	Somewhat Disagree	Somewhat Agree	Strongly Agree	Completely Agree	N/A
My obstetrician asked me how involved in decision making I wanted to be	○	○	○	○	○	○	○
My obstetrician told me that there are different options for my maternity care	○	○	○	○	○	○	○
My obstetrician explained the advantages and disadvantages of the maternity care options	○	○	○	○	○	○	○
My obstetrician helped me understand all the information	○	○	○	○	○	○	○
I was given enough time to thoroughly consider the different care options	○	○	○	○	○	○	○
I was able to choose what I considered to be the best care options	○	○	○	○	○	○	○
My obstetrician respected that choice	○	○	○	○	○	○	○
I deferred to my obstetrician's advice	○	○	○	○	○	○	○

2. Decision making process:

Check the box that responds to your answer

	Completely Disagree	Strongly Disagree	Somewhat Disagree	Somewhat Agree	Strongly Agree	Completely Agree	N/A

My health centre nurse asked me how involved in decision making I wanted to be							
My health centre nurse told me that there are different options for my maternity care							
My health centre nurse explained the advantages and disadvantages of the maternity care options							
My health centre nurse helped me understand all the information							
I was given enough time to thoroughly consider the different care options							
I was able to choose what I considered to be the best care options							
My health centre nurse respected that choice							
I deferred to my health centre nurse's advice							

2. Decision making process:

Check the box that responds to your answer

	Completely Disagree	Strongly Disagree	Somewhat Disagree	Somewhat Agree	Strongly Agree	Completely Agree	N/A
My maternity care provider asked me how involved in decision making I wanted to be							
My maternity care provider told me that there are different options for my maternity care							
My maternity care provider explained the advantages and disadvantages of the maternity care options							
My maternity care provider helped me understand all the information							
I was given enough time to thoroughly consider the different care options							
I was able to choose what I considered to be the best care options							
My maternity care provider respected that choice							

I deferred to my maternity care provider's advice	○	○	○	○	○	○	○

3. I was satisfied with my ability to participate in decision making for my...

Choose all that apply:

- ☐ Pregnancy
- ☐ Labour and birth
- ☐ After the birth
- ☐ Baby care
- ☐ None of the above
- ☐ N/A

4. Overall while making decisions during my pregnancy and birth care I felt:

	Yes	No	N/A
Comfortable asking questions	○	○	○
Comfortable declining care that was offered	○	○	○
Comfortable accepting the options for care that my midwife recommended	○	○	○
Coerced into accepting the options my midwife suggested	○	○	○
I chose the care options that I received	○	○	○
My personal preferences were respected	○	○	○
My cultural preferences were respected	○	○	○

4. Overall while making decisions during my pregnancy and birth care I felt:

	Yes	No	N/A
Comfortable asking questions	○	○	○
Comfortable declining care that was offered	○	○	○
Comfortable accepting the options for care that my family doctor recommended	○	○	○
Coerced into accepting the options my family doctor suggested	○	○	○
I chose the care options that I received	○	○	○
My personal preferences were respected	○	○	○
My cultural preferences were respected	○	○	○

4. Overall while making decisions during my pregnancy and birth care I felt:

	Yes	No	N/A
Comfortable asking questions	○	○	○
Comfortable declining care that was offered	○	○	○
Comfortable accepting the options for care that my obstetrician recommended	○	○	○
Coerced into accepting the options my obstetrician suggested	○	○	○
I chose the care options that I received	○	○	○
My personal preferences were respected	○	○	○

My cultural preferences were respected	○	○	○

4. Overall while making decisions during my pregnancy and birth care I felt:

	Yes	No	N/A
Comfortable asking questions	○	○	○
Comfortable declining care that was offered	○	○	○
Comfortable accepting the options for care that my health centre nurse recommended	○	○	○
Coerced into accepting the options my health centre nurse suggested	○	○	○
I chose the care options that I received	○	○	○
My personal preferences were respected	○	○	○
My cultural preferences were respected	○	○	○

4. Overall while making decisions during my pregnancy and birth care I felt:

	Yes	No	N/A
Comfortable asking questions	○	○	○
Comfortable declining care that was offered	○	○	○
Comfortable accepting the options for care that my maternity care provider recommended	○	○	○
Coerced into accepting the options my maternity care provider suggested	○	○	○
I chose the care options that I received	○	○	○
My personal preferences were respected	○	○	○
My cultural preferences were respected	○	○	○

Please add any additional comments:

Optional

____

5. At any time did you refuse to accept any care that a nurse, doctor or midwife offered to you or your baby? "Care" includes anything that might be done or given to either of you or that you were asked to do (take a test, treatment, medicine, etc.).

- ○ Yes
- ○ No
- ○ Decline to answer
- ○ N/A

Please tell us what you refused, why you refused it, how the staff reacted, and how you felt about it. We would appreciate as much detail as you would care to provide.

____
____
____

5. During this pregnancy, how many times did you expect to see your midwife before birth?

- ○ 4 or more
- ○ Less than 4 (Please explain) ________________________
- ○ Unsure
- ○ N/A

5. During this pregnancy, how many times did you expect to see your family doctor before birth?

- 4 or more
- Less than 4 (Please explain) ________________________
- Unsure
- N/A

5. During this pregnancy, how many times did you expect to see your obstetrician before birth?

- 4 or more
- Less than 4 (Please explain) ________________________
- Unsure
- N/A

5. During this pregnancy, how many times did you expect to see your health centre nurse before birth?

- 4 or more
- Less than 4 (Please explain) ________________________
- Unsure
- N/A

5. During this pregnancy, how many times did you expect to see your maternity care provider before birth?

- 4 or more
- Less than 4 (Please explain) ________________________
- Unsure
- N/A

6. How much time, on average, did you have at prenatal appointments?

- Less than 15 minutes
- 15-30 minutes
- 31-60 minutes
- More than 60 minutes
- N/A

7. I felt that time was:

- Too little
- Just enough
- Too much
- N/A

8. During a prenatal visit, did you ever hold back from asking questions or discussing your concerns because...

	No, never	Yes. once	Yes, more than once	N/A
Your maternity care provider seemed rushed	○	○	○	○
You wanted maternity care that differed from what your maternity care provider recommended	○	○	○	○
You thought your maternity care provider might think you were being difficult	○	○	○	○

9. When you had your baby, how often were you treated poorly by your maternity care provider because of...

	Never	Sometimes	Usually	Always	N/A
Your race, ethnicity, cultural background or language	○	○	○	○	○

Your sexual orientation and/or gender identity	◯	◯	◯	◯	◯
Your health insurance	◯	◯	◯	◯	◯
A difference in opinion with your caregivers about the right care for yourself or your baby	◯	◯	◯	◯	◯

Decision Making: Pregnancy 2 (Optional)

Please describe your experiences with your second care provider making decisions and choosing options for care during this pregnancy.

1. The following answers describe my conversations or experiences with my____________

Please select one:

- Family Doctor
- Obstetrician
- Midwife
- Health Centre Nurse
- Other ________________________

2. Decision making process:

Check the box that responds to your answer

	Completely Disagree	Strongly Disagree	Somewhat Disagree	Somewhat Agree	Strongly Agree	Completely Agree	N/A
My midwife asked me how involved in decision making I wanted to be	○	○	○	○	○	○	○
My midwife told me that there are different options for my maternity care	○	○	○	○	○	○	○
My midwife explained the advantages and disadvantages of the maternity care options	○	○	○	○	○	○	○
My midwife helped me understand all the information	○	○	○	○	○	○	○
I was given enough time to thoroughly consider the different care options	○	○	○	○	○	○	○
I was able to choose what I considered to be the best care options	○	○	○	○	○	○	○
My midwife respected that choice	○	○	○	○	○	○	○
I deferred to my midwife's advice	○	○	○	○	○	○	○

2. Decision making process:

Check the box that responds to your answer

	Completely Disagree	Strongly Disagree	Somewhat Disagree	Somewhat Agree	Strongly Agree	Completely Agree	N/A
My family doctor asked me how involved in decision making I wanted to be	○	○	○	○	○	○	○

My family doctor told me that there are different options for my maternity care	○	○	○	○	○	○	○
My family doctor explained the advantages and disadvantages of the maternity care options	○	○	○	○	○	○	○
My family doctor helped me understand all the information	○	○	○	○	○	○	○
I was given enough time to thoroughly consider the different care options	○	○	○	○	○	○	○
I was able to choose what I considered to be the best care options	○	○	○	○	○	○	○
My family doctor respected that choice	○	○	○	○	○	○	○
I deferred to my family doctor's advice	○	○	○	○	○	○	○

2. Decision making process:

Check the box that responds to your answer

	Completely Disagree	Strongly Disagree	Somewhat Disagree	Somewhat Agree	Strongly Agree	Completely Agree	N/A
My obstetrician asked me how involved in decision making I wanted to be	○	○	○	○	○	○	○
My obstetrician told me that there are different options for my maternity care	○	○	○	○	○	○	○
My obstetrician explained the advantages and disadvantages of the maternity care options	○	○	○	○	○	○	○
My obstetrician helped me understand all the information	○	○	○	○	○	○	○
I was given enough time to thoroughly consider the different care options	○	○	○	○	○	○	○
I was able to choose what I considered to be the best care options	○	○	○	○	○	○	○
My obstetrician respected that choice	○	○	○	○	○	○	○
I deferred to my obstetrician's advice	○	○	○	○	○	○	○

2. Decision making process:

Check the box that responds to your answer

	Completely Disagree	Strongly Disagree	Somewhat Disagree	Somewhat Agree	Strongly Agree	Completely Agree	N/A
My health centre nurse asked me how involved in decision making I wanted to be	○	○	○	○	○	○	○
My health centre nurse told me that there are different options for my maternity care	○	○	○	○	○	○	○
My health centre nurse explained the advantages and disadvantages of the maternity care options	○	○	○	○	○	○	○
My health centre nurse helped me understand all the information	○	○	○	○	○	○	○
I was given enough time to thoroughly consider the different care options	○	○	○	○	○	○	○
I was able to choose what I considered to be the best care options	○	○	○	○	○	○	○
My health centre nurse respected that choice	○	○	○	○	○	○	○
I deferred to my health centre nurse's advice	○	○	○	○	○	○	○

2. Decision making process:

Check the box that responds to your answer

	Completely Disagree	Strongly Disagree	Somewhat Disagree	Somewhat Agree	Strongly Agree	Completely Agree	N/A
My maternity care provider asked me how involved in decision making I wanted to be	○	○	○	○	○	○	○
My maternity care provider told me that there are different options for my maternity care	○	○	○	○	○	○	○
My maternity care provider explained the advantages and disadvantages of the maternity care options	○	○	○	○	○	○	○
My maternity care provider helped me understand all the information	○	○	○	○	○	○	○
I was given enough time to thoroughly consider the different care options	○	○	○	○	○	○	○
I was able to choose what I considered to be the best care options	○	○	○	○	○	○	○

My maternity care provider respected that choice	○	○	○	○	○	○	○
I deferred to my maternity care provider's advice	○	○	○	○	○	○	○

3. I was satisfied with my ability to participate in decision making for my...

Choose all that apply:

- ☐ Pregnancy
- ☐ Labour and birth
- ☐ After the birth
- ☐ Baby care
- ☐ None of the above

4. Overall while making decisions during my pregnancy and birth care I felt:

	Yes	No	N/A
Comfortable asking questions	○	○	○
Comfortable declining care that was offered	○	○	○
Comfortable accepting the options for care that my midwife recommended	○	○	○
Coerced into accepting the options my midwife suggested	○	○	○
I chose the care options that I received	○	○	○
My personal preferences were respected	○	○	○
My cultural preferences were respected	○	○	○

4. Overall while making decisions during my pregnancy and birth care I felt:

	Yes	No	N/A
Comfortable asking questions	○	○	○
Comfortable declining care that was offered	○	○	○
Comfortable accepting the options for care that my family doctor recommended	○	○	○
Coerced into accepting the options my family doctor suggested	○	○	○
I chose the care options that I received	○	○	○
My personal preferences were respected	○	○	○
My cultural preferences were respected	○	○	○

4. Overall while making decisions during my pregnancy and birth care I felt:

	Yes	No	N/A
Comfortable asking questions	○	○	○
Comfortable declining care that was offered	○	○	○
Comfortable accepting the options for care that my obstetrician recommended	○	○	○
Coerced into accepting the options my obstetrician suggested	○	○	○
I chose the care options that I received	○	○	○

My personal preferences were respected	◯	◯	◯
My cultural preferences were respected	◯	◯	◯

4. Overall while making decisions during my pregnancy and birth care I felt:

	Yes	No	N/A
Comfortable asking questions	◯	◯	◯
Comfortable declining care that was offered	◯	◯	◯
Comfortable accepting the options for care that my health centre nurse recommended	◯	◯	◯
Coerced into accepting the options my health centre nurse suggested	◯	◯	◯
I chose the care options that I received	◯	◯	◯
My personal preferences were respected	◯	◯	◯
My cultural preferences were respected	◯	◯	◯

4. Overall while making decisions during my pregnancy and birth care I felt:

	Yes	No	N/A
Comfortable asking questions	◯	◯	◯
Comfortable declining care that was offered	◯	◯	◯
Comfortable accepting the options for care that my maternity care provider recommended	◯	◯	◯
Coerced into accepting the options my maternity care provider suggested	◯	◯	◯
I chose the care options that I received	◯	◯	◯
My personal preferences were respected	◯	◯	◯
My cultural preferences were respected	◯	◯	◯

Please add any additional comments:

Optional

____

5. At any time did you refuse to accept any care that a nurse, doctor or midwife offered to you or your baby? "Care" includes anything that might be done or given to either of you or that you were asked to do (take a test, treatment, medicine, etc.).

◯ Yes
◯ No
◯ Decline to answer

Please tell us what you refused, why you refused it, how the staff reacted, and how you felt about it. We would appreciate as much detail as you would care to provide.

____
____
____

5. During this pregnancy, how many times do you expect to see your midwife before birth?

◯ 4 or more
◯ Less than 4 (Please explain) __________________________
◯ Unsure

5. During this pregnancy, how many times do you expect to see your family doctor before birth?

- 4 or more
- Less than 4 (Please explain) ______________________
- Unsure

5. During this pregnancy, how many times do you expect to see your obstetrician before birth?

- 4 or more
- Less than 4 (Please explain) ______________________
- Unsure

5. During this pregnancy, how many times do you expect to see your health centre nurse before birth?

- 4 or more
- Less than 4 (Please explain) ______________________
- Unsure

5. During this pregnancy, how many times do you expect to see your maternity care provider before birth?

- 4 or more
- Less than 4 (Please explain) ______________________
- Unsure

6. How much time, on average, did you have at prenatal appointments?

- Less than 15 minutes
- 15-30 minutes
- 31-60 minutes
- More than 60 minutes

7. I felt that time was:

- Too little
- Just enough
- Too much

8. During a prenatal visit, did you ever hold back from asking questions or discussing your concerns because...

	No, never	Yes, once	Yes, more than once
Your maternity care provider seemed rushed	○	○	○
You wanted maternity care that differed from what your maternity care provider recommended	○	○	○
You thought your maternity care provider might think you were being difficult	○	○	○

9. When you had your baby, how often were you treated poorly by your maternity care provider because of...

	Never	Sometimes	Usually	Always
Your race, ethnicity, cultural background or language	○	○	○	○
Your sexual orientation and/or gender identity	○	○	○	○
Your health insurance	○	○	○	○
A difference in opinion with your caregivers about the right care for yourself or your baby	○	○	○	○

Decision Making: Pregnancy 2 (Optional)

Please describe your experiences with your third care provider making decisions and choosing options for care during this pregnancy.

1. The following answers describe my conversations or experiences with my____________

Please select one:
- Family Doctor
- Obstetrician
- Midwife
- Health Centre Nurse
- Other ________________________

2. Decision making process:

Check the box that responds to your answer

	Completely Disagree	Strongly Disagree	Somewhat Disagree	Somewhat Agree	Strongly Agree	Completely Agree	N/A
My midwife asked me how involved in decision making I wanted to be							
My midwife told me that there are different options for my maternity care							
My midwife explained the advantages and disadvantages of the maternity care options							
My midwife helped me understand all the information							
I was given enough time to thoroughly consider the different care options							
I was able to choose what I considered to be the best care options							
My midwife respected that choice							
I deferred to my midwife's advice							

2. Decision making process:

Check the box that responds to your answer

	Completely Disagree	Strongly Disagree	Somewhat Disagree	Somewhat Agree	Strongly Agree	Completely Agree	N/A
My family doctor asked me how involved in decision making I wanted to be							

My family doctor told me that there are different options for my maternity care							
My family doctor explained the advantages and disadvantages of the maternity care options							
My family doctor helped me understand all the information							
I was given enough time to thoroughly consider the different care options							
I was able to choose what I considered to be the best care options							
My family doctor respected that choice							
I deferred to my family doctor's advice							

2. Decision making process:

Check the box that responds to your answer

	Completely Disagree	Strongly Disagree	Somewhat Disagree	Somewhat Agree	Strongly Agree	Completely Agree	N/A
My obstetrician asked me how involved in decision making I wanted to be							
My obstetrician told me that there are different options for my maternity care							
My obstetrician explained the advantages and disadvantages of the maternity care options							
My obstetrician helped me understand all the information							
I was given enough time to thoroughly consider the different care options							
I was able to choose what I considered to be the best care options							
My obstetrician respected that choice							
I deferred to my obstetrician's advice							

2. Decision making process:

Check the box that responds to your answer

	Completely Disagree	Strongly Disagree	Somewhat Disagree	Somewhat Agree	Strongly Agree	Completely Agree	N/A
My health centre nurse asked me how involved in decision making I wanted to be							
My health centre nurse told me that there are different options for my maternity care							
My health centre nurse explained the advantages and disadvantages of the maternity care options							
My health centre nurse helped me understand all the information							
I was given enough time to thoroughly consider the different care options							
I was able to choose what I considered to be the best care options							
My health centre nurse respected that choice							
I deferred to my health centre nurse's advice							

2. Decision making process:

Check the box that responds to your answer

	Completely Disagree	Strongly Disagree	Somewhat Disagree	Somewhat Agree	Strongly Agree	Completely Agree	N/A
My maternity care provider asked me how involved in decision making I wanted to be							
My maternity care provider told me that there are different options for my maternity care							
My maternity care provider explained the advantages and disadvantages of the maternity care options							
My maternity care provider helped me understand all the information							
I was given enough time to thoroughly consider the different care options							
I was able to choose what I considered to be the best care options							

My maternity care provider respected that choice							
I deferred to my maternity care provider's advice							

3. I was satisfied with my ability to participate in decision making for my...

Choose all that apply

- Pregnancy
- Labour and birth
- After the birth
- Baby care
- None of the above

4. Overall while making decisions during my pregnancy and birth care I felt:

	Yes	No	N/A
Comfortable asking questions			
Comfortable declining care that was offered			
Comfortable accepting the options for care that my midwife recommended			
Coerced into accepting the options my midwife suggested			
I chose the care options that I received			
My personal preferences were respected			
My cultural preferences were respected			

4. Overall while making decisions during my pregnancy and birth care I felt:

	Yes	No	N/A
Comfortable asking questions			
Comfortable declining care that was offered			
Comfortable accepting the options for care that my family doctor recommended			
Coerced into accepting the options my family doctor suggested			
I chose the care options that I received			
My personal preferences were respected			
My cultural preferences were respected			

4. Overall while making decisions during my pregnancy and birth care I felt:

	Yes	No	N/A
Comfortable asking questions			
Comfortable declining care that was offered			
Comfortable accepting the options for care that my obstetrician recommended			
Coerced into accepting the options my obstetrician suggested			
I chose the care options that I received			

My personal preferences were respected	◯	◯	◯
My cultural preferences were respected	◯	◯	◯

4. Overall while making decisions during my pregnancy and birth care I felt:

	Yes	No	N/A
Comfortable asking questions	◯	◯	◯
Comfortable declining care that was offered	◯	◯	◯
Comfortable accepting the options for care that my health centre nurse recommended	◯	◯	◯
Coerced into accepting the options my health centre nurse suggested	◯	◯	◯
I chose the care options that I received	◯	◯	◯
My personal preferences were respected	◯	◯	◯
My cultural preferences were respected	◯	◯	◯

4. Overall while making decisions during my pregnancy and birth care I felt:

	Yes	No	N/A
Comfortable asking questions	◯	◯	◯
Comfortable declining care that was offered	◯	◯	◯
Comfortable accepting the options for care that my maternity care provider recommended	◯	◯	◯
Coerced into accepting the options my maternity care provider suggested	◯	◯	◯
I chose the care options that I received	◯	◯	◯
My personal preferences were respected	◯	◯	◯
My cultural preferences were respected	◯	◯	◯

Please add any additional comments:

Optional

____

5. At any time did you refuse to accept any care that a nurse, doctor or midwife offered to you or your baby? "Care" includes anything that might be done or given to either of you or that you were asked to do (take a test, treatment, medicine, etc.).

◯ Yes
◯ No
◯ Decline to answer

Please tell us what you refused, why you refused it, how the staff reacted, and how you felt about it. We would appreciate as much detail as you would care to provide.

____
____
____

6. During this pregnancy, how many times do you expect to see your midwife before birth?

◯ 4 or more
◯ Less than 4 (Please explain) __________________________
◯ Unsure

6. During this pregnancy, how many times do you expect to see your family doctor before birth?

- 4 or more
- Less than 4 (Please explain) ________________________
- Unsure

6. During this pregnancy, how many times do you expect to see your obstetrician before birth?

- 4 or more
- Less than 4 (Please explain) ________________________
- Unsure

6. During this pregnancy, how many times do you expect to see your health centre nurse before birth?

- 4 or more
- Less than 4 (Please explain) ________________________
- Unsure

6. During this pregnancy, how many times do you expect to see your maternity care provider before birth?

- 4 or more
- Less than 4 (Please explain) ________________________
- Unsure

7. How much time, on average, did you have at prenatal appointments?

- Less than 15 minutes
- 15-30 minutes
- 31-60 minutes
- More than 60 minutes

8. I felt that time was:

- Too little
- Just enough
- Too much

9. During a prenatal visit, did you ever hold back from asking questions or discussing your concerns because...

	No, never	Yes, once	Yes, more than once
Your maternity care provider seemed rushed	○	○	○
You wanted maternity care that differed from what your maternity care provider recommended	○	○	○
You thought your maternity care provider might think you were being difficult	○	○	○

10. When you had your baby, how often were you treated poorly by your maternity care provider because of...

	Never	Sometimes	Usually	Always
Your race, ethnicity, cultural background or language	○	○	○	○
Your sexual orientation and/or gender identity	○	○	○	○
Your health insurance	○	○	○	○
A difference in opinion with your caregivers about the right care for yourself or your baby	○	○	○	○

Pregnancy History: Currently Pregnant

1. Who is your care provider for this pregnancy?

Please check all that apply. You will have an opportunity to tell us your experiences with up to 3 providers for one pregnancy later on in the survey.

- [ ] Family Doctor
- [ ] Obstetrician
- [ ] Midwife
- [ ] Health Centre Nurse
- [ ] None
- [ ] Other _________________________

If you have no care provider for this pregnancy please tell us why:

____
____
____

If you've had more than one main care provider during this pregnancy please indicate why:

Please check all the apply:

- [ ] Change of provider due to medical concerns
- [ ] Change of provider due to preference
- [ ] Change of provider due to limited maternity services in my community
- [ ] Other, please specify... _________________________

3. Are you expecting twins?

- ( ) Yes
- ( ) No

4. During this pregnancy I experienced:

Please check all that apply:

- [ ] No health problems
- [ ] High blood pressure
- [ ] Depression
- [ ] Problems with baby's growth
- [ ] Problems with baby's health _________________________
- [ ] Diabetes
- [ ] Housing difficulties
- [ ] No support from friends or family
- [ ] Other _________________________

4. During this pregnancy I experienced:

Please check all that apply:

- [ ] No health problems
- [ ] High blood pressure
- [ ] Depression
- [ ] Problems with the babies' growth
- [ ] Problems with the babies' health _________________________
- [ ] Diabetes
- [ ] Housing difficulties
- [ ] No support from friends or family
- [ ] Other _________________________

Decision Making: Currently Pregnant

Please describe your experiences with your first care provider when making decisions and choosing options for care during this pregnancy.

1. The following answers describe my conversations or experiences with my____________

Please select one:
- Family Doctor
- Obstetrician
- Midwife
- Health Centre Nurse
- Other ________________________

2. Decision making process:

Check the box that responds to your answer

	Completely Disagree	Strongly Disagree	Somewhat Disagree	Somewhat Agree	Strongly Agree	Completely Agree	N/A
My midwife asked me how involved in decision making I wanted to be	○	○	○	○	○	○	○
My midwife told me that there are different options for my maternity care	○	○	○	○	○	○	○
My midwife explained the advantages and disadvantages of the maternity care options	○	○	○	○	○	○	○
My midwife helped me understand all the information	○	○	○	○	○	○	○
I was given enough time to thoroughly consider the different care options	○	○	○	○	○	○	○
I was able to choose what I considered to be the best care options	○	○	○	○	○	○	○
My midwife respected that choice	○	○	○	○	○	○	○
I deferred to my midwife's advice	○	○	○	○	○	○	○

2. Decision making process:

Check the box that responds to your answer

	Completely Disagree	Strongly Disagree	Somewhat Disagree	Somewhat Agree	Strongly Agree	Completely Agree	N/A
My family doctor asked me how involved in decision making I wanted to be	○	○	○	○	○	○	○

My family doctor told me that there are different options for my maternity care							
My family doctor explained the advantages and disadvantages of the maternity care options							
My family doctor helped me understand all the information							
I was given enough time to thoroughly consider the different care options							
I was able to choose what I considered to be the best care options							
My family doctor respected that choice							
I deferred to my family doctor's advice							

2. Decision making process:

Check the box that responds to your answer

	Completely Disagree	Strongly Disagree	Somewhat Disagree	Somewhat Agree	Strongly Agree	Completely Agree	N/A
My obstetrician asked me how involved in decision making I wanted to be							
My obstetrician told me that there are different options for my maternity care							
My obstetrician explained the advantages and disadvantages of the maternity care options							
My obstetrician helped me understand all the information							
I was given enough time to thoroughly consider the different care options							
I was able to choose what I considered to be the best care options							
My obstetrician respected that choice							
I deferred to my obstetrician's advice							

2. Decision making process:

Check the box that responds to your answer

	Completely Disagree	Strongly Disagree	Somewhat Disagree	Somewhat Agree	Strongly Agree	Completely Agree	N/A
My health centre nurse asked me how involved in decision making I wanted to be							
My health centre nurse told me that there are different options for my maternity care							
My health centre nurse explained the advantages and disadvantages of the maternity care options							
My health centre nurse helped me understand all the information							
I was given enough time to thoroughly consider the different care options							
I was able to choose what I considered to be the best care options							
My health centre nurse respected that choice							
I deferred to my health centre nurse's advice							

2. Decision making process:

Check the box that responds to your answer

	Completely Disagree	Strongly Disagree	Somewhat Disagree	Somewhat Agree	Strongly Agree	Completely Agree	N/A
My maternity care provider asked me how involved in decision making I wanted to be							
My maternity care provider told me that there are different options for my maternity care							
My maternity care provider explained the advantages and disadvantages of the maternity care options							
My maternity care provider helped me understand all the information							
I was given enough time to thoroughly consider the different care options							
I was able to choose what I considered to be the best care options							

My maternity care provider respected that choice	○	○	○	○	○	○	○
I deferred to my maternity care provider's advice	○	○	○	○	○	○	○

3. I have been satisfied with my ability to participate in decision making.

- ○ Yes
- ○ No

4. Overall while making decisions during this pregnancy I have felt:

	Yes	No	N/A
Comfortable asking questions	○	○	○
Comfortable declining care that is offered	○	○	○
Comfortable accepting options for care recommended by my midwife	○	○	○
Coerced into accepting the options my midwife suggests	○	○	○
I choose the care options that I receive	○	○	○
My personal preferences are respected	○	○	○
My cultural preferences are respected	○	○	○

4. Overall while making decisions during this pregnancy I have felt:

	Yes	No	N/A
Comfortable asking questions	○	○	○
Comfortable declining care that is offered	○	○	○
Comfortable accepting options for care recommended by my family doctor	○	○	○
Coerced into accepting the options my family doctor suggests	○	○	○
I choose the care options that I receive	○	○	○
My personal preferences are respected	○	○	○
My cultural preferences are respected	○	○	○

4. Overall while making decisions during this pregnancy I have felt:

	Yes	No	N/A
Comfortable asking questions	○	○	○
Comfortable declining care that is offered	○	○	○
Comfortable accepting options for care recommended by my obstetrician	○	○	○
Coerced into accepting the options my obstetrician suggests	○	○	○
I choose the care options that I receive	○	○	○
My personal preferences are respected	○	○	○
My cultural preferences are respected	○	○	○

4. Overall while making decisions during this pregnancy I have felt:

	Yes	No	N/A
Comfortable asking questions	◯	◯	◯
Comfortable declining care that is offered	◯	◯	◯
Comfortable accepting options for care recommended by my health centre nurse	◯	◯	◯
Coerced into accepting the options my health centre nurse suggests	◯	◯	◯
I choose the care options that I receive	◯	◯	◯
My personal preferences are respected	◯	◯	◯
My cultural preferences are respected	◯	◯	◯

4. Overall while making decisions during this pregnancy I have felt:

	Yes	No	N/A
Comfortable asking questions	◯	◯	◯
Comfortable declining care that is offered	◯	◯	◯
Comfortable accepting options for care recommended by my maternity care provider	◯	◯	◯
Coerced into accepting the options my maternity care provider suggests	◯	◯	◯
I choose the care options that I receive	◯	◯	◯
My personal preferences are respected	◯	◯	◯
My cultural preferences are respected	◯	◯	◯

Please add any additional comments:

Optional

____

5. At any time did you refuse to accept any care that a nurse, doctor or midwife offered to you or your baby? "Care" includes anything that might be done or given to either of you or that you were asked to do (take a test, treatment, medicine, etc.).

◯ Yes
◯ No
◯ Decline to answer

Please tell us what you refused, why you refused it, how the staff reacted, and how you felt about it. We would appreciate as much detail as you would care to provide.

____
____
____

6. During this pregnancy, how many times do you expect to see your midwife before birth?

◯ 4 or more
◯ Less than 4 (Please explain) __________________________
◯ Unsure

6. During this pregnancy, how many times do you expect to see your family doctor before birth?

◯ 4 or more
◯ Less than 4 (Please explain) __________________________

- Unsure

6. During this pregnancy, how many times do you expect to see your obstetrician before birth?

- 4 or more
- Less than 4 (Please explain) ________________________
- Unsure

6. During this pregnancy, how many times do you expect to see your health centre nurse before birth?

- 4 or more
- Less than 4 (Please explain) ________________________
- Unsure

6. During this pregnancy, how many times do you expect to see your maternity care provider before birth?

- 4 or more
- Less than 4 (Please explain) ________________________
- Unsure

7. How much time, on average, do you have at prenatal appointments?

- Less than 15 minutes
- 15-30 minutes
- 31-60 minutes
- More than 60 minutes

8. I feel that time is:

- Too little
- Just enough
- Too much

9. During a prenatal visit, do you ever hold back from asking questions or discussing your concerns because...

	No, never	Yes, once	Yes, more than once
Your maternity care provider seemed rushed	○	○	○
You wanted maternity care that differed from what your maternity care provider recommended	○	○	○
You thought that your maternity care provider might think you were being difficult	○	○	○

Decision Making: Currently Pregnant (Optional)

Please describe your experiences related to your second care provider making decisions and choosing options for care during this pregnancy.

1. The following answers describe my conversations or experiences with my____________

Please select one:
- ( ) Family Doctor
- ( ) Obstetrician
- ( ) Midwife
- ( ) Health Centre Nurse
- ( ) Other ________________________

2. Decision making process:

Check the box that responds to your answer

	Completely Disagree	Strongly Disagree	Somewhat Disagree	Somewhat Agree	Strongly Agree	Completely Agree	N/A
My midwife asked me how involved in decision making I wanted to be	( )	( )	( )	( )	( )	( )	( )
My midwife told me that there are different options for my maternity care	( )	( )	( )	( )	( )	( )	( )
My midwife explained the advantages and disadvantages of the maternity care options	( )	( )	( )	( )	( )	( )	( )
My midwife helped me understand all the information	( )	( )	( )	( )	( )	( )	( )
I was given enough time to thoroughly consider the different care options	( )	( )	( )	( )	( )	( )	( )
I was able to choose what I considered to be the best care options	( )	( )	( )	( )	( )	( )	( )
My midwife respected that choice	( )	( )	( )	( )	( )	( )	( )
I deferred to my midwife's advice	( )	( )	( )	( )	( )	( )	( )

2. Decision making process:

Check the box that responds to your answer

	Completely Disagree	Strongly Disagree	Somewhat Disagree	Somewhat Agree	Strongly Agree	Completely Agree	N/A
My family doctor asked me how involved in decision making I wanted to be	( )	( )	( )	( )	( )	( )	( )

My family doctor told me that there are different options for my maternity care							
My family doctor explained the advantages and disadvantages of the maternity care options							
My family doctor helped me understand all the information							
I was given enough time to thoroughly consider the different care options							
I was able to choose what I considered to be the best care options							
My family doctor respected that choice							
I deferred to my family doctor's advice							

2. Decision making process:

Check the box that responds to your answer

	Completely Disagree	Strongly Disagree	Somewhat Disagree	Somewhat Agree	Strongly Agree	Completely Agree	N/A
My obstetrician asked me how involved in decision making I wanted to be							
My obstetrician told me that there are different options for my maternity care							
My obstetrician explained the advantages and disadvantages of the maternity care options							
My obstetrician helped me understand all the information							
I was given enough time to thoroughly consider the different care options							
I was able to choose what I considered to be the best care options							
My obstetrician respected that choice							
I deferred to my obstetrician's advice							

2. Decision making process:

Check the box that responds to your answer

	Completely Disagree	Strongly Disagree	Somewhat Disagree	Somewhat Agree	Strongly Agree	Completely Agree	N/A
My health centre nurse asked me how involved in decision making I wanted to be	○	○	○	○	○	○	○
My health centre nurse told me that there are different options for my maternity care	○	○	○	○	○	○	○
My health centre nurse explained the advantages and disadvantages of the maternity care options	○	○	○	○	○	○	○
My health centre nurse helped me understand all the information	○	○	○	○	○	○	○
I was given enough time to thoroughly consider the different care options	○	○	○	○	○	○	○
I was able to choose what I considered to be the best care options	○	○	○	○	○	○	○
My health centre nurse respected that choice	○	○	○	○	○	○	○
I deferred to my health centre nurse's advice	○	○	○	○	○	○	○

2. Decision making process:

Check the box that responds to your answer

	Completely Disagree	Strongly Disagree	Somewhat Disagree	Somewhat Agree	Strongly Agree	Completely Agree	N/A
My maternity care provider asked me how involved in decision making I wanted to be	○	○	○	○	○	○	○
My maternity care provider told me that there are different options for my maternity care	○	○	○	○	○	○	○
My maternity care provider explained the advantages and disadvantages of the maternity care options	○	○	○	○	○	○	○
My maternity care provider helped me understand all the information	○	○	○	○	○	○	○
I was given enough time to thoroughly consider the different care options	○	○	○	○	○	○	○
I was able to choose what I considered to be the best care options	○	○	○	○	○	○	○

My maternity care provider respected that choice							
I deferred to my maternity care provider's advice							

3. I have been satisfied with my ability to participate in decision making for my...

Choose all that apply:
- Pregnancy
- Labour and birth
- After the birth
- Baby care
- None of the above

4. Overall while making decisions during this pregnancy I have felt:

	Yes	No	N/A
Comfortable asking questions			
Comfortable declining care that is offered			
Comfortable accepting options for care recommended by my midwife			
Coerced into accepting the options my midwife suggests			
I choose the care options that I receive			
My personal preferences are respected			
My cultural preferences are respected			

4. Overall while making decisions during this pregnancy I have felt:

	Yes	No	N/A
Comfortable asking questions			
Comfortable declining care that is offered			
Comfortable accepting options for care recommended by my family doctor			
Coerced into accepting the options my family doctor suggests			
I choose the care options that I receive			
My personal preferences are respected			
My cultural preferences are respected			

4. Overall while making decisions during this pregnancy I have felt:

	Yes	No	N/A
Comfortable asking questions			
Comfortable declining care that is offered			
Comfortable accepting options for care recommended by my obstetrician			
Coerced into accepting the options my obstetrician suggests			
I choose the care options that I receive			

My personal preferences are respected	◯	◯	◯
My cultural preferences are respected	◯	◯	◯

4. Overall while making decisions during this pregnancy I have felt:

	Yes	No	N/A
Comfortable asking questions	◯	◯	◯
Comfortable declining care that is offered	◯	◯	◯
Comfortable accepting options for care recommended by my health centre nurse	◯	◯	◯
Coerced into accepting the options my health centre nurse suggests	◯	◯	◯
I choose the care options that I receive	◯	◯	◯
My personal preferences are respected	◯	◯	◯
My cultural preferences are respected	◯	◯	◯

4. Overall while making decisions during this pregnancy I have felt:

	Yes	No	N/A
Comfortable asking questions	◯	◯	◯
Comfortable declining care that is offered	◯	◯	◯
Comfortable accepting options for care recommended by my maternity care provider	◯	◯	◯
Coerced into accepting the options my maternity care provider suggests	◯	◯	◯
I choose the care options that I receive	◯	◯	◯
My personal preferences are respected	◯	◯	◯
My cultural preferences are respected	◯	◯	◯

Please add any additional comments:

Optional

____

5. At any time did you refuse to accept any care that a nurse, doctor or midwife offered to you or your baby? "Care" includes anything that might be done or given to either of you or that you were asked to do (take a test, treatment, medicine, etc.).

◯ Yes
◯ No
◯ Decline to answer

Please tell us what you refused, why you refused it, how the staff reacted, and how you felt about it. We would appreciate as much detail as you would care to provide.

____
____
____

6. During this pregnancy, how many times do you expect to see your midwife before birth?

◯ 4 or more
◯ Less than 4 (Please explain) __________________________
◯ Unsure

6. During this pregnancy, how many times do you expect to see your family doctor before birth?

- 4 or more
- Less than 4 (Please explain) ________________________
- Unsure

6. During this pregnancy, how many times do you expect to see your obstetrician before birth?

- 4 or more
- Less than 4 (Please explain) ________________________
- Unsure

6. During this pregnancy, how many times do you expect to see your community health nurse before birth?

- 4 or more
- Less than 4 (Please explain) ________________________
- Unsure

6. During this pregnancy, how many times do you expect to see your maternity care provider before birth?

- 4 or more
- Less than 4 (Please explain) ________________________
- Unsure

7. How much time, on average, do you have at prenatal appointments?

- Less than 15 minutes
- 15-30 minutes
- 31-60 minutes
- More than 60 minutes

8. I feel that time is:

- Too little
- Just enough
- Too much

9. During a prenatal visit, do you ever hold back from asking questions or discussing your concerns because...

	No, never	Yes, once	Yes, more than once
Your maternity care provider seemed rushed	○	○	○
You wanted maternity care that differed from what your maternity care provider recommended	○	○	○
You thought that your maternity care provider might think you were being difficult	○	○	○

Decision Making: Currently Pregnant (Optional)

Please describe your experiences related to your third care provider making decisions and choosing options for care during this pregnancy.

1. The following answers describe my conversations or experiences with my____________

Please select one:
- Family Doctor
- Obstetrician
- Midwife
- Health Centre Nurse
- Other ________________________

2. Decision making process:

Check the box that responds to your answer

	Completely Disagree	Strongly Disagree	Somewhat Disagree	Somewhat Agree	Strongly Agree	Completely Agree	N/A
My midwife asked me how involved in decision making I wanted to be							
My midwife told me that there are different options for my maternity care							
My midwife explained the advantages and disadvantages of the maternity care options							
My midwife helped me understand all the information							
I was given enough time to thoroughly consider the different care options							
I was able to choose what I considered to be the best care options							
My midwife respected that choice							
I deferred to my midwife's advice							

2. Decision making process:

Check the box that responds to your answer

	Completely Disagree	Strongly Disagree	Somewhat Disagree	Somewhat Agree	Strongly Agree	Completely Agree	N/A
My family doctor asked me how involved in decision making I wanted to be							

My family doctor told me that there are different options for my maternity care							
My family doctor explained the advantages and disadvantages of the maternity care options							
My family doctor helped me understand all the information							
I was given enough time to thoroughly consider the different care options							
I was able to choose what I considered to be the best care options							
My family doctor respected that choice							
I deferred to my family doctor's advice							

2. Decision making process:

Check the box that responds to your answer

	Completely Disagree	Strongly Disagree	Somewhat Disagree	Somewhat Agree	Strongly Agree	Completely Agree	N/A
My obstetrician asked me how involved in decision making I wanted to be							
My obstetrician told me that there are different options for my maternity care							
My obstetrician explained the advantages and disadvantages of the maternity care options							
My obstetrician helped me understand all the information							
I was given enough time to thoroughly consider the different care options							
I was able to choose what I considered to be the best care options							
My obstetrician respected that choice							
I deferred to my obstetrician's advice							

2. Decision making process:

Check the box that responds to your answer

	Completely Disagree	Strongly Disagree	Somewhat Disagree	Somewhat Agree	Strongly Agree	Completely Agree	N/A
My health centre nurse asked me how involved in decision making I wanted to be							
My health centre nurse told me that there are different options for my maternity care							
My health centre nurse explained the advantages and disadvantages of the maternity care options							
My health centre nurse helped me understand all the information							
I was given enough time to thoroughly consider the different care options							
I was able to choose what I considered to be the best care options							
My health centre nurse respected that choice							
I deferred to my health centre nurse's advice							

2. Decision making process:

Check the box that responds to your answer

	Completely Disagree	Strongly Disagree	Somewhat Disagree	Somewhat Agree	Strongly Agree	Completely Agree
My maternity care provider asked me how involved in decision making I wanted to be						
My maternity care provider told me that there are different options for my maternity care						
My maternity care provider explained the advantages and disadvantages of the maternity care options						
My maternity care provider helped me understand all the information						
I was given enough time to thoroughly consider the different care options						
I was able to choose what I considered to be the best care options						

My maternity care provider respected that choice	○	○	○	○	○	○
I deferred to my maternity care provider's advice	○	○	○	○	○	○

3. I have been satisfied with my ability to participate in decision making for my...

Choose all that apply:

- ☐ Pregnancy
- ☐ Labour and birth
- ☐ After the birth
- ☐ Baby care
- ☐ None of the above

4. Overall while making decisions during this pregnancy I have felt:

Check all that apply:

	Yes	No	N/A
Comfortable asking questions	○	○	○
Comfortable declining care that is offered	○	○	○
Comfortable accepting options for care recommended by my midwife	○	○	○
Coerced into accepting the options my midwife suggests	○	○	○
I choose the care options that I receive	○	○	○
My personal preferences are respected	○	○	○
My cultural preferences are respected	○	○	○

4. Overall while making decisions during this pregnancy I have felt:

Check all that apply:

	Yes	No	N/A
Comfortable asking questions	○	○	○
Comfortable declining care that is offered	○	○	○
Comfortable accepting options for care recommended by my family doctor	○	○	○
Coerced into accepting the options my family doctor suggests	○	○	○
I choose the care options that I receive	○	○	○
My personal preferences are respected	○	○	○
My cultural preferences are respected	○	○	○

4. Overall while making decisions during this pregnancy I have felt:

Check all that apply:

	Yes	No	N/A
Comfortable asking questions	○	○	○
Comfortable declining care that is offered	○	○	○
Comfortable accepting options for care recommended by my obstetrician	○	○	○

Coerced into accepting the options my obstetrician suggests	◯	◯	◯
I choose the care options that I receive	◯	◯	◯
My personal preferences are respected	◯	◯	◯
My cultural preferences are respected	◯	◯	◯

4. Overall while making decisions during this pregnancy I have felt:

Check all that apply:

	Yes	No	N/A
Comfortable asking questions	◯	◯	◯
Comfortable declining care that is offered	◯	◯	◯
Comfortable accepting options for care recommended by my health centre nurse	◯	◯	◯
Coerced into accepting the options my health centre nurse suggests	◯	◯	◯
I choose the care options that I receive	◯	◯	◯
My personal preferences are respected	◯	◯	◯
My cultural preferences are respected	◯	◯	◯

4. Overall while making decisions during this pregnancy I have felt:

Check all that apply:

	Yes	No	N/A
Comfortable asking questions	◯	◯	◯
Comfortable declining care that is offered	◯	◯	◯
Comfortable accepting options for care recommended by my maternity care provider	◯	◯	◯
Coerced into accepting the options my maternity care provider suggests	◯	◯	◯
I choose the care options that I receive	◯	◯	◯
My personal preferences are respected	◯	◯	◯
My cultural preferences are respected	◯	◯	◯

Please add any additional comments:

Optional

____

5. At any time did you refuse to accept any care that a nurse, doctor or midwife offered to you or your baby? "Care" includes anything that might be done or given to either of you or that you were asked to do (take a test, treatment, medicine, etc.).

◯ Yes
◯ No
◯ Decline to answer

Please tell us what you refused, why you refused it, how the staff reacted, and how you felt about it. We would appreciate as much detail as you would care to provide.

____

____

6. During this pregnancy, how many times do you expect to see your midwife before birth?

- 4 or more
- Less than 4 (Please explain) ________________________
- Unsure

6. During this pregnancy, how many times do you expect to see your family doctor before birth?

- 4 or more
- Less than 4 (Please explain) ________________________
- Unsure

6. During this pregnancy, how many times do you expect to see your obstetrician before birth?

- 4 or more
- Less than 4 (Please explain) ________________________
- Unsure

6. During this pregnancy, how many times do you expect to see your community health nurse before birth?

- 4 or more
- Less than 4 (Please explain) ________________________
- Unsure

6. During this pregnancy, how many times do you expect to see your maternity care provider before birth?

- 4 or more
- Less than 4 (Please explain) ________________________
- Unsure

7. How much time, on average, do you have at prenatal appointments?

- Less than 15 minutes
- 15-30 minutes
- 31-60 minutes
- More than 60 minutes

8. I feel that time is:

- Too little
- Just enough
- Too much

9. During a prenatal visit, do you ever hold back from asking questions or discussing your concerns because...

	No, never	Yes, once	Yes, more than once
Your maternity care provider seemed rushed	○	○	○
You wanted maternity care that differed from what your maternity care provider recommended	○	○	○
You thought that your maternity care provider might think you were being difficult	○	○	○

Travelling for Care

This section asks questions to help us understand how far families have to go to access the care they want.

1. What is your postal code?

____

2. Which community do you live in?

____

3. I was able to easily find a doctor to care for me.

- Yes
- No
- N/A

4. I was able to easily find a midwife to care for me.

- Yes
- No
- N/A

If no to either of the above, please check all that apply:

- No pregnancy doctor in my community
- No midwife in my community
- No doctor accepting new patients
- No midwife accepting new patients
- Pregnancy deemed high risk
- Distance
- Cost
- Other, please specify... _________________________

5. Did you and/or your partner have to travel more than 60 minutes to access maternity care during labour?

- Yes
- No
- N/A

If yes, please tell us when you travelled?

Check all that apply:

- During my third trimester, before labour started
- I left my community in my ________ week of pregnancy _________________________
- During labour

6. Did you travel outside of your community to have a home birth (at a hotel or a friend's house)?

- Yes
- No

Additional Comments (Optional):

____
____
____

7. What kind of impact did travelling for care have on you and your family?

- Positive

- [ ] Negative
- [ ] Neutral
- [ ] N/A

Additional Comments (Optional):

____

Travelling for Care

This section asks questions to help us understand how far people will go to access the care they want.

1. What is your postal code?

____

2. Which community do you live in?

____

3. I expect to be able to easily find a doctor to care for me.

- ○ Yes
- ○ No
- ○ N/A

4. I expect to be able to easily find a midwife to care for me.

- ○ Yes
- ○ No
- ○ N/A

If no to either of the above, please check all that apply:

- ☐ No pregnancy doctor in my community
- ☐ No midwife in my community
- ☐ No doctor accepting new patients
- ☐ No midwife accepting new patients
- ☐ Pregnancy deemed high risk
- ☐ Distance
- ☐ Cost
- ☐ Other, please specify... ____________________________

5. Will you and/or your partner have to travel more than 60 minutes to access maternity care during labour?

- ○ Yes
- ○ No
- ○ I don't know

6. Would you travel outside of your community to have a home birth (at a hotel or a friend's house)?

- ○ Yes
- ○ No

Additional Comments (Optional):

____
____
____

7. What kind of impact do you expect travelling for care would have on you and your family?

- ☐ Positive
- ☐ Negative
- ☐ Neutral
- ☐ N/A

Additional Comments (Optional):

Midwifery Care

1. In your own words, please describe what a midwife is and/or does?

____
____
____

2. The next section is about what people understand about midwives in British Columbia.

	Agree	Disagree	Not Sure
Midwives are legal in BC	◯	◯	◯
Midwives deliver babies (attend births) only in the home	◯	◯	◯
Midwives deliver babies (attend births) only in the hospital	◯	◯	◯
Midwives deliver babies (attend births) both in homes and hospitals	◯	◯	◯
Midwives provide pregnancy care only	◯	◯	◯
Midwives provide pregnancy and after birth care but do not attend births	◯	◯	◯
Midwives provide pregnancy, birth and after birth care	◯	◯	◯
Midwives care for the baby after the birth for 6-8 weeks	◯	◯	◯
Midwives can order prenatal tests (ultrasounds, blood work)	◯	◯	◯
Midwives can order medicines during pregnancy	◯	◯	◯
Midwives use natural methods to promote comfort and progress in labour (e.g. water, herbs, massage)	◯	◯	◯
Midwives use medicines to promote comfort and progress in labour	◯	◯	◯
Midwives stay with the woman throughout labour and birth	◯	◯	◯
Midwives do home visits	◯	◯	◯
Midwives help with breastfeeding	◯	◯	◯
Midwives work under doctors	◯	◯	◯
Midwives offer family planning services	◯	◯	◯
A midwife office visit is usually 30-60 minutes long	◯	◯	◯
A midwife who attends a birth has usually also cared for the woman during her pregnancy	◯	◯	◯
Midwives only care for women with no health problems	◯	◯	◯
Midwives can treat some pregnancy and birth problems	◯	◯	◯
Midwives work with an obstetrician when a woman has serious pregnancy health problems	◯	◯	◯
Family doctors work with an obstetrician when a woman has serious health problems	◯	◯	◯
Midwives do women's health exams (e.g. pap smears)	◯	◯	◯

3. Midwifery care is:

- Paid for privately
- Covered by BC's Medical Services Plan
- Don't know

4. If I had another pregnancy I would choose a:

- Family Doctor
- Obstetrician
- Midwife
- Other ________________________

5. If a friend or family member were to become pregnant I would recommend that she find care from a:

- Family Doctor
- Obstetrician
- Midwife
- Other ________________________

6. I know how to find a midwife:

- Yes
- No

7. I would use the following to find a midwife:

Check all the apply:

- [ ] Through a website
- [ ] Word of mouth
- [ ] Through a blog
- [ ] Google search
- [ ] Asking doctor or nurse
- [ ] Friends
- [ ] Other ________________________

Your Background

All answers to this survey are confidential. The questions in this section help our researchers understand the overall background of people who answer the survey. We will only report results for groups.

1. How old are you?

____

2. Do you identify as First Nations, Inuit or Métis?

- Yes
- No

3. I identify myself as:

Check all that apply:
- Arab (Middle Eastern)
- Black (African, Caribbean, etc.)
- Caucasian (White, European)
- Chinese
- Filipino/a
- Japanese
- Korean
- Latin American
- South Asian (Indian, Pakistani, Sri Lankan)
- Southeast Asian (Cambodian, Indonesian, Laotian, Vietnamese)
- West Asian (Afghani, Iranian)
- Other (Please specify) ___________________________
- I prefer not to answer

4. Please tell us your category of family income:

Please estimate
• Less than $30,000
• $31,000-$50,000
• $51,000-$70,000
• $71,000-$90,000
• $91,000 or more
• I prefer not to answer

5. What is your highest level of education?

• Elementary school
• High school
• College
• Undergraduate degree
• Graduate degree
• Postgraduate degree
• Prefer not to answer

Thank you very much for your interest in the study, but you do not meet the eligibility criteria for participation.

Additional Comments

1. Is there anything else that you would like to add? Or is there anything important that we forgot to ask?

____
____
____

2. We will be arranging forums to further discuss some of these topics. Are you interested in being contacted about participating in a future forum?

- ◯ Yes
- ◯ No

Please provide your email address.
Note: Your information will only be used to contact you regarding focus group participation.

____

Thank you for taking part!

Please feel free to invite other women to complete the survey by sending them this link:

http://fluidsurveys.com/s/changingchildbirthinbc_survey1/
